# Clinical correlates of blood-derived circulating tumor DNA in pancreatic cancer

**DOI:** 10.1186/s13045-019-0824-4

**Published:** 2019-12-04

**Authors:** Hitendra Patel, Ryosuke Okamura, Paul Fanta, Charmi Patel, Richard B. Lanman, Victoria M. Raymond, Shumei Kato, Razelle Kurzrock

**Affiliations:** 1 Center for Personalized Cancer Therapy and Division of Hematology and Oncology, Department of Medicine, UC San Diego Moores Cancer Center, 3855 Health Sciences Drive, La Jolla, CA 92093 USA; 2 Department of Pathology, UC San Diego, La Jolla, CA USA; 3 Department of Medical Affairs, Guardant Health, Inc., Redwood City, CA USA

**Keywords:** Pancreatic cancer, Next-generation sequencing, Circulating tumor DNA, KRAS, Molecular oncology, Targeted therapy

## Abstract

**Background:**

Treatment outcomes for patients with advanced pancreatic ductal adenocarcinoma (PDAC) remain dismal. There are unmet needs for understanding the biologic basis of this malignancy using novel next-generation sequencing technologies. Herein, we investigated the clinical utility of circulating tumor DNA (ctDNA) (the liquid biopsy) in this malignancy.

**Methods:**

ctDNA was analyzed in 112 patients with PDAC (54–73 genes) and tissue DNA in 66 patients (315 genes) (both clinical-grade next-generation sequencing). Number of alterations, %ctDNA, concordance between ctDNA and tissue DNA, and correlation of ctDNA results with survival were assessed.

**Results:**

The most common genes altered in ctDNA were *TP53* (46% of patients, *N* = 51) and *KRAS* (44%, *N* = 49). Median number of characterized ctDNA alterations per patient was 1 (range, 0–6), but patients with advanced PDAC had significantly higher numbers of ctDNA alterations than those with surgically resectable disease (median, 2 versus 0.5, *P* = 0.04). Overall, 75% (70/94) of advanced tumors had ≥ 1 ctDNA alteration. Concordance rate between ctDNA and tissue DNA alterations was 61% for *TP53* and 52% for *KRAS*. Concordance for *KRAS* alterations between ctDNA and tissue DNA from metastatic sites was significantly higher than between ctDNA and primary tumor DNA (72% vs 39%, *P* = 0.01). Importantly, higher levels of total %ctDNA were an independent prognostic factor for worse survival (hazard ratio, 4.35; 95% confidence interval, 1.85–10.24 [multivariate, *P* = 0.001]). A patient with three ctDNA alterations affecting the MEK pathway (*GNAS*, *KRAS*, and *NF1*) attained a response to trametinib monotherapy ongoing at 6 months.

**Conclusions:**

Our findings showed that ctDNA often harbored unique alterations some of which may be targetable and that significantly greater numbers of ctDNA alterations occur in advanced versus resectable disease. Furthermore, higher ctDNA levels were a poor prognostic factor for survival.

## Introduction

In the USA, there are approximately 56,770 people newly diagnosed with pancreatic cancer in 2019, with high mortality (~ 46,000 deaths) [1–3] .Although the mortality from other types of gastrointestinal cancer, such as gastric or colorectal cancers, are declining over the past two decades, mortality from pancreatic cancer has not declined [1]. It is estimated that pancreatic cancer will become the second cause of cancer death by 2030 [2].

The majority of pancreatic cancers (80–90%) are classified as pancreatic ductal adenocarcinomas (PDACs) [3]. One of the underlying reasons for high mortality associated with PDACs is that most patients have late-stage disease at the time of diagnosis. In fact, only 15–20% of patients are considered to be surgical candidates at the diagnosis [4]. Furthermore, prognosis even among patients who were able to have surgery with negative margins remains poor (5-year survival rate was only 10–25% with the median survival between 10-20 months) [5, 6].

Multi-agent systemic chemotherapies with regimens of 5-fluorouracil, leucovorin, irinotecan, and oxaliplatin (FOLFIRINOX) and gemcitabine plus nab-paclitaxel have shown improved survival over single-agent gemcitabine and have become standard treatment options for metastatic PDACs [7, 8]. However, median progression-free survival (PFS) and overall survival (OS) remain dismal (PFS: FOLFIRINOX, 6.4 months; gemcitabine plus nab-paclitaxel, 5.5 months, gemcitabine alone, 3.3–3.7 months; OS: FOLFIRINOX, 11.1 months; gemcitabine plus nab-paclitaxel, 8.5 months, gemcitabine alone, 6.7–6.8 months) [7, 8]. Therefore, along with the recent development in sequencing technology, a personalized, molecular-targeted approach for PDAC is becoming an active area of research [9]. Several case studies showed a benefit of platinum-based chemotherapies or poly ADP-ribose polymerase (PARP) inhibitors for PDAC patients with *BRCA1/2* abnormalities, and the NCCN guidelines suggest consideration of platinum-based regimen as a first-line therapy for advanced-stage pancreatic cancer patients with *BRCA* gene mutations [10–14].

Although molecular analysis on tissue samples is generally attempted, its clinical utility is often diminished in pancreatic cancer due to the difficulty in obtaining tissue with adequate quality for comprehensive molecular testing [15]. Furthermore, tumor heterogeneity may challenge small biopsies, particularly in metastatic disease with multiple tumors [16]. In contrast, the utility of plasma-derived circulating tumor DNA (ctDNA) has recently been assessed in several tumor types [17–21]. ctDNA has some advantages over tissue DNA analysis: (1) readily available, (2) less-invasive, (3) potential real-time monitoring of disease status or treatment response, and (4) may reflect shed DNA from multiple metastatic sites [22–24]. On the other hand, the small amount of tumor DNA in the circulation results in limitations as well.

Herein, we assessed the genomic landscape of ctDNA in patients with PDAC, using clinical-grade next-generation sequencing (NGS). We investigated the clinical implications of the findings including concordance between tissue and blood DNA sequencing, relationship between ctDNA findings and survival, and potential as well as actual actionability, with the latter illustrated by a patient with multiple alterations affecting the MEK pathway whose tumor responded to the MEK inhibitor trametinib.

## Materials and methods

### Study population

We reviewed the clinicopathological and genomic information of 112 consecutive eligible patients with PDAC who had a blood-derived ctDNA evaluation. Only patients with pathologically proven PDAC were included. All investigations followed the guidelines of the University of California San Diego Moores Cancer Center Internal Review Board and were performed in accordance with the Declaration of Helsinki under the auspices of our approved study Profile-Related Evidence Determining Individualized Cancer Therapy study (PREDICT study, NCT02478931) and any investigational therapy for which the patients gave consent [25].

### Circulating tumor DNA (ctDNA) and tissue DNA sequencing

#### ctDNA NGS

All blood samples for ctDNA were evaluated at a clinical laboratory improvement amendments (CLIA) licensed and College of American Pathologist (CAP) accredited clinical laboratory, *Guardant Health, Inc*. (Redwood City, CA. http://www.guardanthealth.com). Blood samples were collected in 10 ml Streck tubes, and 5 to 30 ng of ctDNA was used for sequencing as previously described [26]. The ctDNA assay can sequence cancer-associated genes to identify somatic alterations with high analytic sensitivity, which reaches detection of 1–2 single mutant fragments from a 10-mL blood sample (0.1% limit of detection) and high specificity (> 99.9999%) (54 to 73 genes, Additional file 1: Table S1). Only characterized genomic alterations were used for analysis (synonymous alterations or variants of unknown significance were excluded). Mutant allele frequency (%ctDNA) was calculated as the number of mutant molecules divided by the total number of DNA fragments in each mutated gene. Percent ctDNA could not be calculated for gene amplifications. This study considered %ctDNA for only characterized alterations and used median value as a cutoff for maximum %ctDNA (of any alteration in a patient) or total %ctDNA (evaluating all alterations per patient).

#### Tissue DNA NGS

All tissue DNA analyses in this study were performed by a CLIA-licensed and CAP-accredited laboratory, *Foundation Medicine, Inc.* (Cambridge, MA. https://www.foundationmedicine.com). The sequencing was designed to include all genes somatically altered in human solid malignancies that were validated as targets for therapy, either approved or in clinical trials, and/or that were unambiguous drivers of oncogenesis based on available knowledge. The assay interrogated 315 genes [27, 28].

### Actionable alterations in ctDNA

This study assessed actionability for each genomic alteration in ctDNA. We defined a characterized alteration as potentially druggable if it (or its pathway signal) could be impacted at low inhibitory concentrations for small molecule inhibitors or if an antibody specific to the protein product of the alteration impacted it. Only cognate agents approved by the US. Food and Drug Administration (FDA) (on- or off-label use) or compounds that are currently in clinical trials were considered (Additional file 1: Table S2).

### Outcomes and statistics

The continuous variables (described with median value and range) and categorical variables (described with frequency and percentage) were compared with the Mann-Whitney *U* test and Fisher’s exact test, respectively. In terms of ctDNA results according to disease stage, we compared metastatic, locally advanced, or recurrent (abbreviated as “advanced”) disease setting with surgical resectable cases whose blood was biopsied before radical surgery. Concordance of genomic alterations between ctDNA and tissue DNA was assessed by concordance rate with *Kappa* value. *Kappa* value is interpreted by commonly used agreement categories: 1 (perfect agreement) to 0 (no agreement other than would be expected by chance). Most common gene aberrations (altered in ≥ 8 samples) were used for concordance analysis. When patients were stratified according by tissue biopsy site or time interval between blood draw for ctDNA and tissue biopsy, the difference in concordance rate was compared by Fisher’s exact test. OS time was measured from date of blood draw for ctDNA to date of last follow-up or all-cause death. In order to explore independent prognostic factors for the OS, we used Cox’s proportional hazard model in multivariate analysis to estimate hazard ratio (HR) with 95% confidence interval (CI). Variables with *P* < 0.1 in the univariate analyses (using the log-rank test) were entered into the multivariate analysis. RO performed and verified statistical analysis using SPSS version 24 software (*IBM Corporation*, Armonk, NY).

## Results

### Genomic alterations in ctDNA for PDAC

**Fig. 1:**
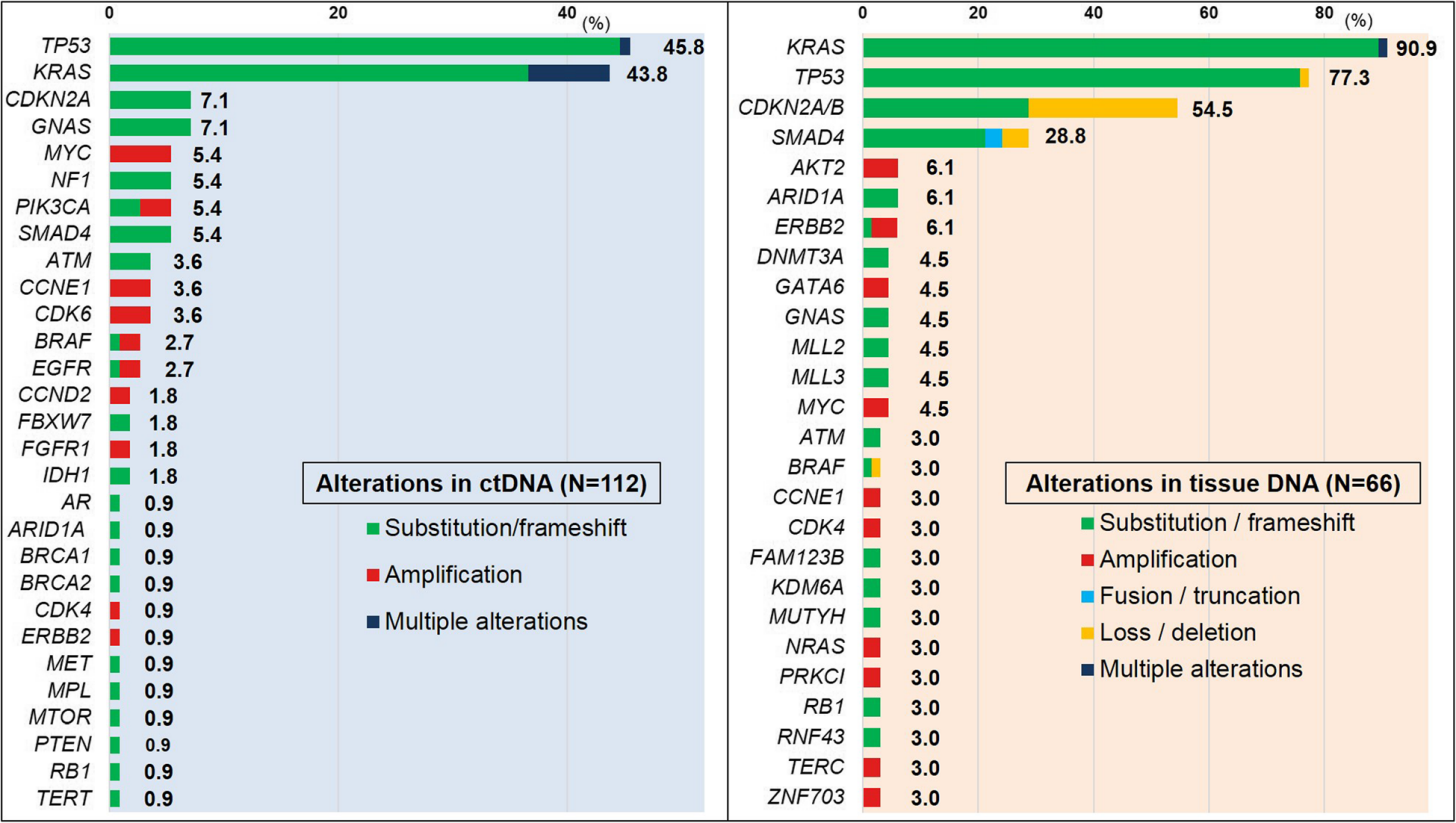
Frequency (% of patients) of characterized alterations in pancreatic ductal adenocarcinoma (ctDNA [*N* = 112] and tissue DNA [*N* = 66]). Only genes altered in ≥ 2 patients were shown in tissue DNA

A total of 112 patients with PDAC were evaluated for ctDNA. Among them, 94 patients (84%) were analyzed in the advanced setting, while 18 patients (16%) were analyzed before (*N* = 10) or after radical surgery (*N* = 8) for lesions that were surgically resectable at the time of blood draw for ctDNA (Table 1). Of the 112 patients, 70% (*N* = 78) had ≥ 1 characterized alteration in ctDNA, and the median number of characterized alterations per patient was 1 (range, 0 to 6). A total of 194 characterized alterations were identified, including 158 amino acid substitutions or frameshift mutations (81%), 35 gene amplifications (18%), and 1 gene deletion (0.5%). These characterized alterations occurred in 29 different genes and included 112 distinct alterations. The most common genes altered were *TP53* (46% of patients, *N* = 51)*, KRAS* (44%, *N* = 49), *CDKN2A* (7%, *N* = 8), and *GNAS* (7%, *N* = 8) (Fig. 1).

**Table 1:** Patient characteristics and number of genomic alterations detected in ctDNA among pancreatic ductal adenocarcinoma patients (*N* = 112)

Characteristics for all patients [*N* = 112]		*N* (%)	
Median age (range) (years)*		67.8 (38.0–92.7)	
Gender			
Men		60 (53.6%)	
Women		52 (46.4%)	
Ethnicity			
Caucasian		76 (67.9%)	
Hispanic		16 (14.3%)	
Asian		7 (6.3%)	
African American		4 (3.6%)	
Other/unknown		9 (8.0%)	
Disease status at the time of blood draw			
Advanced (metastatic, locally advanced or recurrent disease)		94 (83.9%)	
Preoperative (surgically resectable, blood obtained before surgery)		10 (8.9%)	
Postoperative (surgically resectable, blood obtained after surgery)		8 (7.1%)	
Number of patients with ≥ 1 characterized alteration		78 (69.6%)	
Median number of characterized alterations per patient (range)		1 (0–6)	
Comparisons of ctDNA results between advanced cases and preoperative cases**			
Parameters	Advanced cases (*N* = 94)	Preoperative cases (*N* = 10)	*P* value
Number of characterized alterations per patient (range), median (range) (%)	2 (0–6)	0.5 (0–3)	**0.04**
No. of patients with detectable characterized alterations	70 (74.5%)	5 (50.0%)	0.14
Maximum %ctDNA per patient (characterized alterations), median (range) (%)	0.4 (0.0–64.6)	0.0 (0.0–0.62)	**0.02**
Total %ctDNA per patient (characterized alterations), median (range) (%)	0.6 (0.0 - 92.05)	0.0 (0.0 - 0.70)	**0.007**

*Age at date of advanced disease diagnosis or date of first diagnosis for surgically resectable diseases

**Postoperative cases (blood obtained after radical surgery) were not included in these comparisons

#### Most patients had ctDNA alterations that were potentially pharmacologically tractable

Overall, 90% of the characterized alterations (175/194) in ctDNA were potentially targetable with FDA-approved agents (on- or off-label use), and an additional 3% (6/194) were theoretically targetable with therapies that are currently in clinical trials (Additional file 1: Table S2). Meanwhile, 68% (*N* = 76) of the 112 patients had ≥ 1 theoretically actionable alterations by an FDA-approved agent (on- or off-label). Furthermore, among the 94 patients with advanced PDAC, 73% (*N* = 69) had ≥ 1 theoretically actionable alteration by FDA-approved agents (on- or off-label).

#### Patients with PDAC mostly had ctDNA portfolios that were distinct at the molecular level despite common alterations at the genomic level

Among the 78 patients who had ≥ 1 characterized alteration, ID#60 and ID#88 (*KRAS* G12 V and *KRAS* amplification), ID#24 and ID#102 (*TP53* V216 M), ID#8 and ID#85 (*KRAS* G12R), and ID#64 and ID#70 (*KRAS* G12 V) had molecularly identical portfolios in ctDNA. In addition, two patients harbored alterations in *TP53*, *KRAS*, and *CDKN2A* genes (ID#7 and ID#51), and 10 patients harbored alterations in *TP53* and *KRAS* genes (ID#3, ID#14, ID#31, ID#74, ID#76, ID#87, ID#95, ID#96, ID#101, and ID#108), reflecting genomic portfolios that were identical but alterations at the level of the locus that were different (Additional file 1: Table S3).

#### Patients with advanced PDAC had more ctDNA alterations and higher %ctDNA than those with surgically resectable disease

When evaluated depending on disease stage at the time of blood draw, median number of characterized alterations per patient was significantly higher in the patients with advanced PDAC (*N* = 94) than those with surgically resectable disease (*N* = 10) (the blood for ctDNA was biopsied before surgery) (2 versus 0.5, *P* = 0.04) (Table 1). Overall, 75% of patients with advanced PDAC had ≥ 1 characterized alteration, compared with 50% of patients with surgically resectable PDACs (*P* = 0.14). Moreover, the maximum %ctDNA (among the characterized alterations) per patient was significantly higher in the patients with advanced PDAC than those with surgically resectable PDACs (median, 0.4 versus 0.0, *P* = 0.02), as well as the total %ctDNA per patient (median, 0.6 versus 0.0, *P* = 0.007). Complete list of ctDNA alterations and disease status at the time of ctDNA analysis for all the patients was shown in Additional file 1: Table S4. When comparing the ctDNA parameters according to disease stage at the time of ctDNA analysis among the 94 patients with advanced PDAC (between patients with ≥ 1 treatment regimen prior to ctDNA test and chemotherapy naïve), no significant differences were observed (Additional file 1: Table S5).

### Concordance of altered genes between ctDNA and tissue DNA sequencing

Of the 112 patients, 66 patients (59%) also had tissue DNA NGS analysis (Additional file 1: Figure S1). In tissue DNA, alterations were commonly observed in *KRAS*, (91% of the patients, *N* = 60), *TP53* (77%, *N* = 51), and *CDKN2A/B* (55%, *N* = 36) (Fig. 1). Among the 66 patients, only four patients (6.1%) had genomically concordant results between ctDNA and tissue DNA (Additional file 1: Table S6), and an additional 31 patients (47%) had partially concordant results (having ≥ 1 identical gene mutation) between the ctDNA and tissue sequencing.

Overall genomic concordance rate between ctDNA and tissue DNA analyses was 61% for *TP53* and 52% for *KRAS* (Table 2). Although the alteration rate in *CDKN2A* was 7% (*N* = 8 of 112 samples) in ctDNA versus 55% (*N* = 36 of 66 samples) in tissue (Fig. 1), this discrepancy might be attributable to the fact that earlier versions of the ctDNA panel did not assess allelic loss in this gene; hence concordance rate was not assessable for the *CDKN2A* gene.

**Table 2:** Overall concordance between ctDNA and tissue DNA based on time interval between blood draw and tissue biopsy and on whether primary or metastatic tumor was biopsied (*N* = 66)

Patients with pancreatic ductal adenocarcinoma who had both ctDNA and tissue DNA sequencing (*N* = 66)										
		Tissue DNA (+)		Tissue DNA (−)		Overall concordance* (%)	Kappa (SE)			
*TP53*	ctDNA (+)	29 (44%)		4 (6.1%)		61	0.21 (0.1)			
	ctDNA (−)	22 (33%)		11 (17%)						
*KRAS*	ctDNA (+)	28 (42%)		0 (0.0%)		52	0.14 (0.1)			
	ctDNA (−)	32 (48%)		6 (9.1%)						
Concordance depending on whether primary tumor or metastatic site was biopsied										
		Primary tumor (*n* = 41)				Metastatic sites (*n* = 25)				
		Tissue DNA (+)	Tissue DNA (−)	Overall concordance* (%)	Kappa (SE)	Tissue DNA (+)	Tissue DNA (−)	Overall concordance* (%)	Kappa (SE)	*P* value
*TP53*	ctDNA (+)	14 (34%)	1 (2.4%)	54	0.19 (0.1)	15 (60%)	3 (12%)	72	0.27 (0.2)	0.20
	ctDNA (−)	18 (44%)	8 (20%)			4 (16%)	3 (12%)			
*KRAS*	ctDNA (+)	14 (34%)	0 (0.0%)	39	0.05 (0.04)	14 (56%)	0 (0.0%)	72	0.39 (0.2)	0.01
	ctDNA (−)	25 (61%)	2 (4.9%)			7 (28%)	4 (16%)			
Concordance based on time interval between blood draw and tissue biopsy										
		≤ 6 months (*n* = 49)				>6 months (*n* = 17)				
		Tissue DNA (+)	Tissue DNA (−)	Overall concordance* (%)	Kappa (SE)	Tissue DNA (+)	Tissue DNA (−)	Overall concordance* (%)	Kappa (SE)	*P* value
*TP53*	ctDNA (+)	25 (51%)	3 (6.1%)	65	0.24 (0.1)	4 (24%)	1 (5.9%)	47	0.10 (0.2)	0.25
	ctDNA (−)	14 (29%)	7 (14%)			8 (47%)	4 (24%)			
*KRAS*	ctDNA (+)	22 (45%)	0 (0.0%)	55	0.17 (0.1)	6 (35%)	0 (0.0%)	41	0.07 (0.1)	0.40
	ctDNA (−)	22 (45%)	5 (10%)			10 (59%)	1 (6%)			

*Genomic concordance was analyzed in this table rather than molecular locus concordance

#### Spatial and temporal effects on concordance

When compared according to tissue biopsy site (primary tumor versus metastatic sites), the concordance rate for *KRAS* was significantly higher between ctDNA and metastatic sites than between ctDNA and primary tumor (72% versus 39%, *P* = 0.01); the difference for *TP53* was not significant (Table 2). When compared according to the time interval between blood draw and tissue biopsy, the concordance rates for *TP53* and *KRAS* genes were numerically higher in patients with ≤ 6 months of time interval than those with > 6 months (55–65% versus 41–47%, but not statistically significant). Furthermore, consistent concordance rates for *TP53* and *KRAS* alterations were observed among different ctDNA sequencing panels (when we compared detection rates for *TP53* and *KRAS* alterations according to ctDNA panel [73-gene panel, *N* = 41, versus 54–70-gene panels, *N* = 25], there were no significant differences (*P* = 0.61 for *TP53* and *P* > 0.99 for *KRAS* alterations).

### Overall survival from ctDNA analysis among patients with advanced PDAC

### Higher levels of ctDNA were associated with shorter survival

**Fig. 2:**
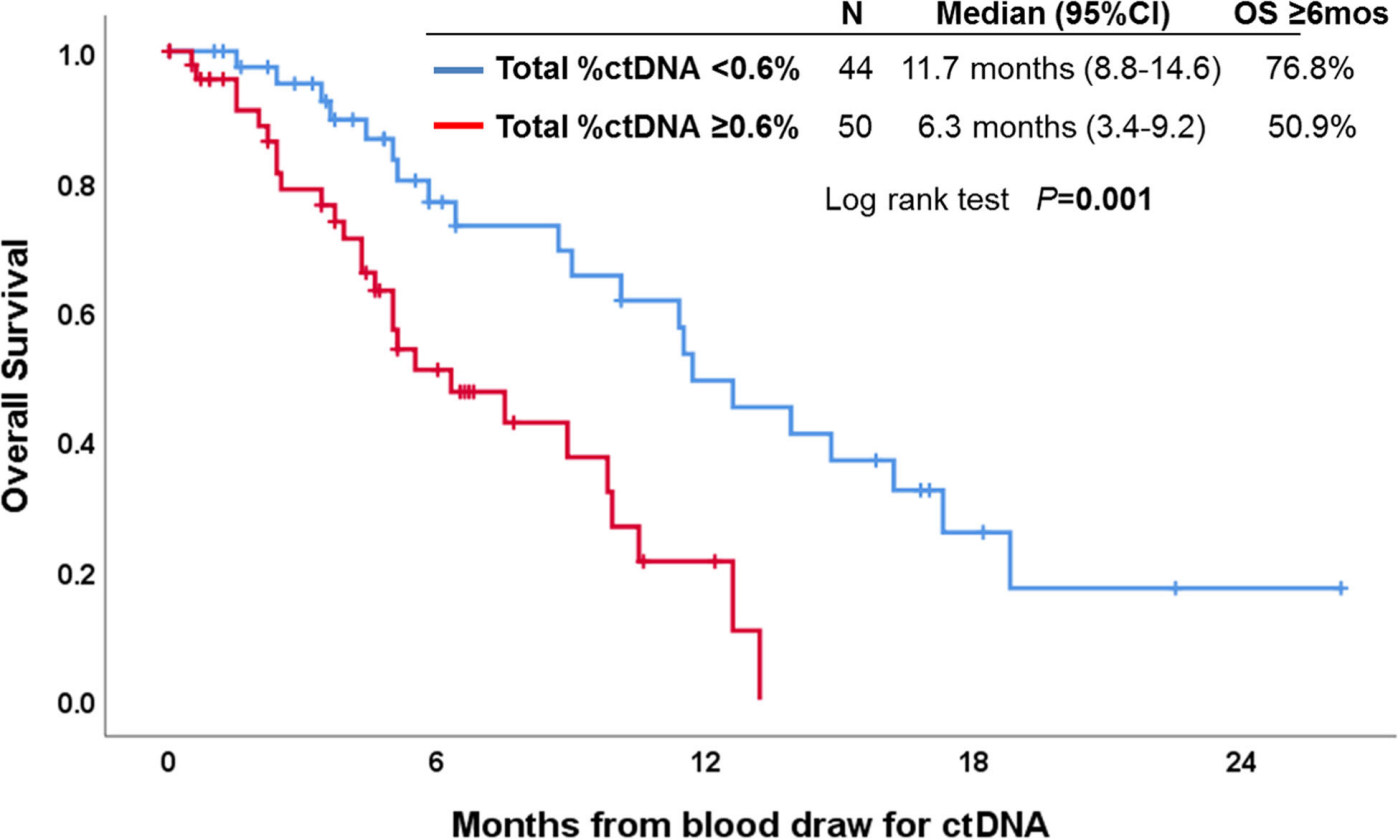
Kaplan-Meier curve for overall survival from ctDNA analysis depending on total %ctDNA (characterized alterations only) dichotomized at the median among patients with advanced pancreatic ductal adenocarcinoma (*N* = 94)

Survival analysis included 94 patients with advanced PDAC in whom the median follow-up time was 18.2 months (95% CI, 13.7–22.7). In the univariate analysis, the presence of *KRAS* alterations in ctDNA and higher level of total %ctDNA (≥ 0.6% [0.6% being the median %ctDNA when total ctDNA was assessed]) were associated with worse OS (Table 3 and Fig. 2). When the variables with *P* value < 0.1 were entered into the multivariate analysis, higher level of total %ctDNA (HR 4.35, 95% CI 1.85–10.24) (*P* = 0.001) and ≥ 1 systemic therapy prior to ctDNA analysis (HR 2.89, 95% CI 1.51–5.55) (*P* = 0.001) were significantly associated with worse OS from ctDNA analysis in PDAC (OS from advanced disease diagnosis was also shorter in patients with higher level of total %ctDNA) (Additional file 1: Figure S2). Even when the patients were stratified by ctDNA sequencing panel, there was no major difference between the 73-gene panel and the 54-70-gene panels (Additional file 1 Figure S3). Presence of *KRAS o*r *TP53* alteration in ctDNA was not significantly associated with OS in the multivariate analysis.

**Table 3:** Multivariate analysis for factors associated with overall survival from date of ctDNA analysis in patients with advanced PDAC (*N* = 94)

Characteristics	Univariate analysis		Multivariate analysis*	
	Median OS months	*P* value	HR (95%CI)	*P* value
Age				
≥ 68 (*N* = 47) vs < 68 (*N* = 47)	11.5 vs 9.0	0.77	–	–
Gender				
Men (*N* = 51) vs women (*N* = 43)	9.9 vs 8.9	0.25	–	–
Genomic alterations in ctDNA				
*KRAS* (*N* = 48) vs not (*N* = 46)	7.5 vs 11.4	**0.03**	1.14 (0.53–2.45)	0.74
*TP53* (*N* = 45) vs not (*N* = 49)	8.9 vs 10.1	0.67		
Maximum %ctDNA**				
≥ 0.4% (*N* = 49) vs < 0.4% (*N* = 45)	8.9 vs 11.4	0.17	–	–
Total %ctDNA**				
≥ 0.6% (*N* = 50) vs < 0.6% (*N* = 44)	6.3 vs 11.7	**0.001**	4.35 (1.85–10.24)	**0.001**
Number of characterized alterations				
≥ 1 (*N* = 70) vs none (*N* = 24)	8.9 vs 11.4	0.27	–	–
Number of systemic therapies prior to ctDNA analysis				
≥ 1 regimen (*N* = 40) vs none (*N* = 54)	6.4 vs 9.9	0.09	2.89 (1.51–5.55)	**0.001**

*Abbreviations*: *CI* confidence interval, *ctDNA* circulating tumor DNA, *HR* hazard ratio; *%ctDNA* mutant allele frequency, *OS* overall survival

*Factors with *P* value < 0.1 in univariate analysis were included in the multivariate analysis

**Only characterized alterations were considered (synonymous alteration and VUS were excluded). Dichotomized at the median of 0.4% for the maximum %ctDNA and 0.6% for the total %ctDNA

### ctDNA analysis and selection of targeted therapy

Among the 94 patients with advanced PDAC, only 8 patients had treatment initiated based on the ctDNA results, and one of these patients responded (13%). This patient was unusual in that he had three alterations in the MEK pathway (discussed below).

#### Patient ID#111

**Fig. 3:**
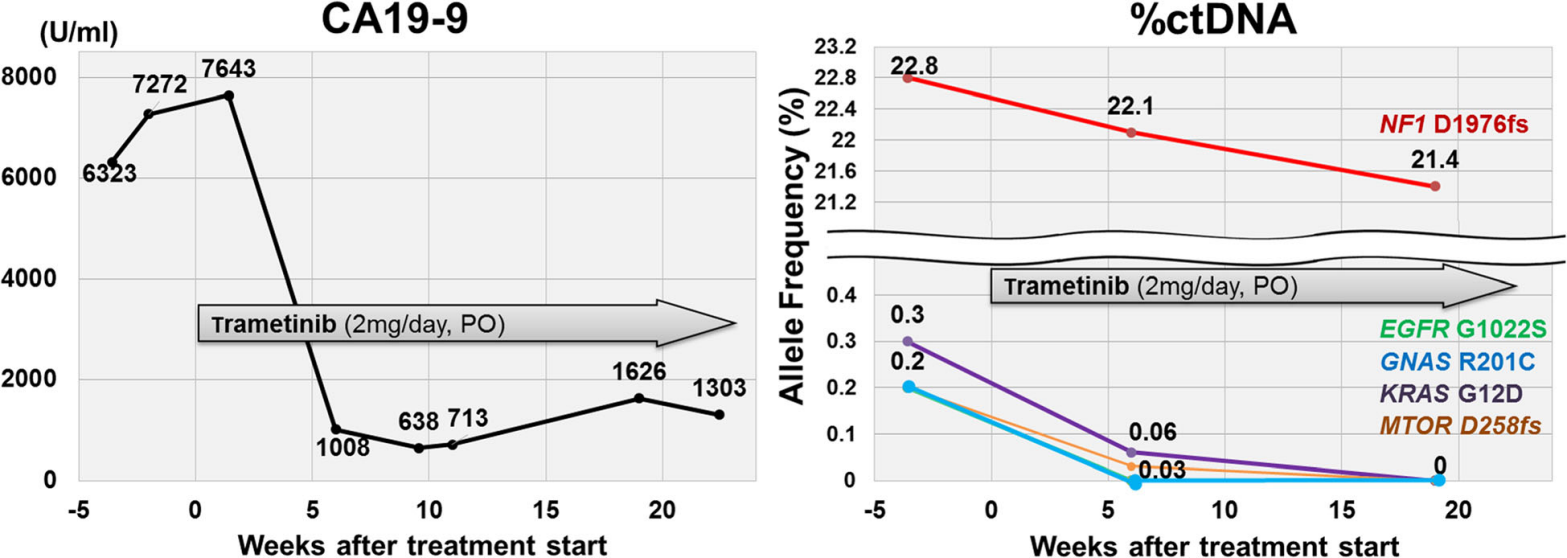
A representative PDAC case who underwent a matched targeted therapy based on ctDNA analysis. An 83-year-old man treated with the MEK inhibitor trametinib as single-agent therapy [ID#111]; he had alterations in *GNAS*, *KRAS,* and *NF1*, all of which can activate the MEK pathway. The patient showed improvement in symptoms, CA19-9, and %ctDNA in the altered genes. CT scans with contrast could not be performed due to renal insufficiency. Treatment is ongoing at 26+ weeks

An 83-year-old man with a locally advanced PDAC presented with abdominal and back pain (Eastern Cooperative Oncology Group Performance Status [ECOG-PS] = 2). His CT scan showed a pancreatic body tumor (6.5 × 3.2 cm) spreading to the retroperitoneum and encasing the aorta and celiac artery and the dilation of the pancreatic duct. The biliary obstruction was not observed. Unfortunately, tissue biopsy of the pancreatic mass was insufficient for genomic analysis. However, his blood-derived ctDNA revealed *EGFR* G1022S, *GNAS* R201C, *KRAS* G12D, *MTOR* D258fs, and *NF1* D1976fs. Because of the patient’s age, he was hesitant to undergo chemotherapy. Based on the ctDNA analysis, he started the first-line treatment with trametinib (potentially targeting *GNAS, KRAS*, and *NF1* alterations (Additional file 1: Table S2) [29–34]. He tolerated this treatment well and his pain significantly improved. Although his restaging CT was not evaluable due to inability to use iodine contrast (underlying renal insufficiency), his PET-CT at 15 weeks showed only mild uptake in the primary tumor and CA19-9 demonstrated a remarkable decrease from 7272 to 1303 U/ml (normal upper limit is 42 U/ml) (Fig. 3). Serial ctDNA was analyzed at 19 weeks after the initiation of treatment and showed a decrease in %ctDNA for *NF1*, and *EGFR*, *GNAS, KRAS*, and *MTOR* mutations were no longer detected (Fig. 3). The treatment continues at 26+ weeks with good tolerance.

## Discussion

Despite the recent development of aggressive chemotherapies, patients with pancreatic cancer, who are generally diagnosed with advanced stage disease, have a dismal outcome. Therefore, improvement in treatment strategies for this lethal malignancy based on a better understanding of its biology is urgently needed. Here, we investigated the landscape of ctDNA NGS in pancreatic cancer, its concordance with tissue DNA NGS, and the clinical implications of the findings.

We found that 70% of patients with PDAC had ≥ 1 characterized genomic alteration in ctDNA (Table 1). Importantly, among the 94 patients with advanced PDAC, 73% (*N* = 69) had ≥ 1 theoretically actionable alteration by FDA-approved agents (on- or off-label) (Additional file 1: Table S2), opening the doors for precision matching trials [35–37]. These observations may be especially pertinent because several approved agents (such as gemcitabine and erlotinib) have only a small impact on survival [38]. The weak clinical impact may be due to lack of biomarker selection when prescribing treatment or existence of multiple oncogenic alterations. In fact, our data also showed that patients with advanced PDAC mostly had a unique pattern of molecular portfolios in ctDNA (even when the actual genomic alterations overlapped) and that more than half of them (*N* = 48/94) had two or more alterations, suggesting the need for a deep understanding of the effect of abnormalities in specific gene loci [39]. For instance, *KRAS* is well known as the dominant oncogene in pancreatic cancer and its prevalence is generally over 90% [40, 41]. Consistently, our data showed the prevalence of *KRAS* alterations was 91% in tissue DNA NGS (Fig. 1). Meanwhile, 9.1% (*N* = 6/66) of patients with ctDNA and tissue DNA NGS had *KRAS* wild type in both of the two tests (Table 2). Several previous reports have suggested unique targetable alterations even in tumors with *KRAS* wild type, such as *EGFR* exon 19 deletion and *ERBB2* amplification [15, 42]. In our series, among the six patients whose ctDNA and tissue DNA NGS were both *KRAS* wild type, all had at least one theoretically actionable alteration in both the two tests, including *EGFR* amplification or *ERBB2* amplification.

Not unexpectedly, characterized alterations in ctDNA were more frequent in patients with advanced PDAC than in those with surgically resectable disease (median, 2 versus 0.5, *P* = 0.04); median of maximum %ctDNA (0.4% versus 0%, *P* = 0.02) and median of total %ctDNA (0.6% versus 0%, *P* = 0.007) were also higher (Table 1). These findings are consistent with a previous report showing that ctDNA was more easily detectable in patients with metastatic cancer than those with localized diseases [43, 44]. Higher tumor load presumably increases ctDNA shedding to blood.

Overall concordance rate between ctDNA and tissue DNA was 61% for *TP53* anomalies and 52% for *KRAS* alterations (Table 2). In this series, the frequency of alterations in each gene was lower in ctDNA than in tissue DNA (Fig. 1). It should be noted that the sensitivity of ctDNA for tissue DNA in detecting alterations was lower, compared with that of tissue DNA for ctDNA (57% [29 of 51] versus 88% [29 of 33] for *TP53*, and 47% [28 of 60] versus 100% [28 of 28] for *KRAS*, respectively). Other studies have found similar results [45–47]. Discordant cases that were positive in tissue and negative in ctDNA have been previously explained by low tumor load in surgically resectable cases [43, 44]. In addition, detection of ctDNA can be affected by systemic treatment prior to blood draw [48, 49]. In terms of spatial effects on concordance, we demonstrated that *KRAS* concordance was significantly higher between ctDNA and metastatic sites than between ctDNA and primary tumor (72% versus 39%, *P* = 0.01) (Table 2). Consistent with our observations, a prior study evaluating heterogeneity in ctDNA genomic profiling results in gastroesophageal cancers also reported that several targetable genomic alterations were 88% concordant between metastatic tissue and ctDNA even when primary tumor and metastatic sites had discordant results [23]. The authors suggested that biomarker profiling of metastatic site tissue or ctDNA was potentially more effective in selection of therapy than interrogating primary sites. In fact, in this series, ctDNA *TP53* and *KRAS* alteration concordance rates in the patients whose tissues were biopsied from metastatic sites were numerically higher than the rates in the patients whose tissues were biopsied from primary tumors (72% versus 54% for *TP53*, *P* = 0.20; 72% versus 39% for *KRAS*, *P* = 0.01) (Table 2). (The rate of tissue *TP53* alterations was 76% [19 of 25] for metastatic sites and 78% [32 of 41] for primary sites; for *KRAS* alterations, it was 84% [21 of 25] versus 95% [39 of 41]; hence, there was no increased frequency of either alteration in tissue from metastatic sites.) It is conceivable that patients who have visible metastatic tumor that can be biopsied for sequencing may have higher tumor burden than those whose tissues for sequencing were available only from primary tumor, and this may explain the higher concordance rate with ctDNA. Further investigation is required [47, 50, 51]. Somewhat surprisingly, there was no statistically significant difference in concordance when ctDNA and tissue sampling dates were ≤ 6 months versus > 6 months apart. To further assess these spatial and temporal effects on concordance, larger numbers of samples are required.

We also report that the total %ctDNA (dichotomized at median %ctDNA) was associated with patient survival (median OS from blood draw for ctDNA, 6.3 versus 11.7 months, *P* = 0.001; median OS from advanced disease diagnosis: 10.8 versus 18.2 months, *P =* 0.03) (Fig. 2 and Additional file 1: Figure S2). Several studies previously reported that the presence of detectable ctDNA was associated with poor survival in pancreatic cancer [52, 53] or that the presence of *KRAS* alterations in ctDNA was a poor prognostic marker for OS in advanced PDAC [52, 54, 55]. In our series, multivariate analysis showed that ≥1 prior therapy and higher total %ctDNA, the latter perhaps reflecting greater tumor burden or shedding potential, were independently associated with worse OS (for higher total %ctDNA, HR, 4.35; 95%CI, 1.85–10.24; multivariate *P* = 0.001) (Table 3). Also, the presence of prior therapies (HR, 2.89; 95%CI, 1.51–5.55; multivariate *P* = 0.001) may reflect refractory cases who may have poorer prognosis.

To date, accumulating evidence has shown that matching drugs to sequenced genomic alterations can be promising for patients with advanced cancer [56–59]. However, we were only able to match eight patients to therapy based on ctDNA and only one (13%) showed salutary effects (Fig. 3). The patient is unusual in several ways. In general, it is known that single targeted agents have limited effects in pancreatic cancer [31, 60, 61]. Tumor heterogeneity or the existence of co-alterations may mediate resistance to scripted monotherapies [62]. However, our patient had multiple alterations that could activate the MEK pathway (*GNAS* R201C, *KRAS* G12D, and *NF1* D1976fs) [29–33] and demonstrated a remarkable responsiveness to the MEK inhibitor trametinib, with a steep decline in CA19-9 and %ctDNA as well as improvement in symptoms and PET imaging after therapy showing only minimal uptake in the tumor (Fig. 3). Our observation differs from previous literature suggesting that MEK inhibitors lack substantial anti-tumor activity among patients with pancreatic cancer [31, 63]. The salutary effects in our patient might be due to the multiple MEK pathway abnormalities harbored by his cancer.

Meanwhile, low target-drug matching rate in this series (8 of 94 patients with advanced PDAC) is a realistic challenge. The remaining 55 patients received unmatched conventional chemotherapies following the molecular profiling and 31 had no systemic chemotherapies following the molecular profiling (mostly due to clinical deterioration or continuation of the regimen prior to the ctDNA test). Also, many patients with pancreatic cancer have genomic alterations that are not considered easily druggable. Therefore, to further investigate the efficacy of matched targeted therapy approaches, improvement in drug or clinical trial access as well as ctDNA testing in patients with less advanced disease will be necessary.

In the NCCN guidelines (https://www.nccn.org), testing for germline and somatic *BRCA1/2* alterations is recommended for selection of platinum-based chemotherapies or PARP inhibitors based on emerging data from several small studies [11, 12, 64–66]. In our series, the prevalence of *BRCA1/2* abnormalities in ctDNA and tissue DNA NGS were 1.8% (*N* = 2/112; *BRCA1* Splice Site SNV and *BRCA2* T3033 fs) and 3.0% (*N* = 2/66; *BRCA1* truncation intron 16 and *BRCA2* A938fs*21), respectively (germline alterations were not captured). Several genomic alterations are rare and the number of patients who can benefit from targeting those individual abnormalities is small, but further study to investigate highly targetable biomarkers based on deep sequencing can be justified.

This study has several limitations. First, the ctDNA gene panel expanded with time, increasing from 54 to 73 genes (Additional file 1: Table S1). Therefore, a limitation of the study pertains to the fact that the sequencing panels were different and so not all genes sequenced in tissue were sequenced in ctDNA. Nonetheless, our tissue and ctDNA panels allowed the comparison of most of the commonly altered genes in pancreatic cancer using clinical-grade assays frequently utilized in patients. The discrepancy in the frequency of *CDKN2A/B* loss between ctDNA and tissue (with lower frequency in ctDNA) probably results from the fact that its allelic loss was not captured in older panels of the ctDNA sequencing. Second, not all patients had both ctDNA and tissue DNA tests; therefore, future concordance analysis should be performed with larger numbers of patients. Moreover, further analysis with tissue DNA from both primary tissue and metastatic sites may help inform the issues related to intratumoral heterogeneity (though in many patients with pancreatic cancer, accessing biopsy sites can be challenging or dangerous). Third, analysis of the influence of systemic treatment on ctDNA alterations is not feasible in this study due to the lack of serial ctDNA testing per patient. Finally, additional studies are also needed to determine the impact of matching ctDNA alterations to therapy beyond the eight patients matched in the current investigation.

## Conclusions

The majority of patients with PDAC (70%) had at least one characterized genomic alteration and over 40% had *TP53* or *KRAS* alterations in ctDNA (Table 1 and Fig. 1). Among patients with advanced PDAC, 73% had at least one alteration that is potentially pharmacologically tractable by FDA-approved agents. Patients with advanced PDAC had higher numbers of characterized alterations and higher maximum and total %ctDNA when compared to those with surgically resectable diseases (Table 1). ctDNA results for *KRAS* mutations were significantly more concordant with tissue DNA when the biopsy was from a metastatic site (versus the primary site) (Table 2). Detection of higher levels of total %ctDNA was an independent prognostic factor for poor overall survival (Fig. 2 and Table 3). A unique patient with three different alterations affecting the MEK pathway showed a sustained response to the MEK inhibitor trametinib, indicating that the nuances of how to best match patients to cognate agents merit further study in PDAC.

## Supplementary information

**Additional file 1:** **Tables S1**–**S6** and **Figures S1**–**S3** Supplementary tables and figures.

## Data Availability

The dataset used and analyzed during the current study are available from the corresponding author on reasonable request.

## Abbreviations

%ctDNA: Mutant allele frequency
CAP: College of American Pathologist
CI: Confidence interval
CLIA: Clinical laboratory improvement amendments
ctDNA: Circulating tumor DNA
ECOG-PS: Eastern Cooperative Oncology Group Performance Status
FDA: Food and Drug Administration of the United States
FOLFIRINOX: A combination of 5-fluorouracil, leucovorin, irinotecan, and oxaliplatin
HR: Hazard ratio
NGS: Next-generation sequencing
OS: Overall survival
PARP: Poly ADP-ribose polymerase
PDAC: Pancreatic ductal adenocarcinoma
PFS: Progression-free survival
PREDICT study: Profile-Related Evidence Determining Individualized Cancer Therapy study
