# Supplementary material for: Clinical correlates of blood-derived circulating tumor DNA in pancreatic cancer
Source: J Hematol Oncol. 2019 Dec 4;12:130. doi: 10.1186/s13045-019-0824-4 (PMC6894333; doi:10.1186/s13045-019-0824-4)
Supplement: Supplementary file 1 — Additional file 1: Tables S1–S6 and Figures S1–S3 Supplementary tables and figures. [file 13045_2019_824_MOESM1_ESM.docx]

**SUPPLEMENTARY MATERIALS**

**Supplementary Table 1.** 54 to 73 gene panels of ctDNA (*Guardant, Inc*.)

**Supplementary Table 1a.** 54 gene panel (N=3 samples).

| **POINT MUTATIONS** | | | | **AMPLIFICATIONS** |
| --- | --- | --- | --- | --- |
| *ABL1* | *AKT1* | ***ALK*** | ***APC*** | *EGFR* |
| ***AR*** | *ATM* | ***BRAF*** | ***CDKN2A*** | *ERBB2* |
| *CDH1* | *CSF1R* | *CTNNB1* | ***EGFR*** | *MET* |
| ***ERBB2*** | *ERBB4* | *EZH2* | ***FBXW7*** |  |
| *FGFR1* | *FGFR2* | *FGFR3* | *FLT3* |  |
| *GNA11* | *GNAQ* | *GNAS* | *HNF1A* |  |
| *HRAS* | *IDH1* | *IDH2* | *JAK2* |  |
| *JAK3* | *KDR* | *KIT* | ***KRAS*** |  |
| ***MET*** | *MLH1* | *MPL* | ***MYC*** |  |
| ***NOTCH1*** | *NPM1* | ***NRAS*** | *PDGFRA* |  |
| ***PIK3CA*** | ***PTEN*** | *PTPN11* | ***PROC*** |  |
| ***RB1*** | *RET* | *SMAD4* | *SMARCB1* |  |
| *SMO* | *SRC* | *STK11* | *TERT* |  |
| ***TP53*** | *VHL* |  |  |  |

All exons were sequenced in genes in ***bold***.

**Supplementary Table 1b.** 68 gene panel (N=18 samples).

| **POINT MUTATIONS** | | | | **AMPLIFICATIONS** | **FUSIONS** | **INDELS** |
| --- | --- | --- | --- | --- | --- | --- |
| *AKT1* | *ALK* | ***APC*** | ***AR*** | *AR* | *ALK* | *EGFR* exon 19 deletions |
| *AFAR* | ***ARID1A*** | *ATM* | ***BRAF*** | *BRAF* | *NTRK1* | *EGFR* exon 20 insertions |
| ***BRCA1*** | ***BRCA2*** | ***CCDN1*** | ***CCDN2*** | *CCNE1* | *RET* |  |
| ***CCNE1*** | *CDH1* | ***CDK4*** | ***CDK6*** | *CDK4* | *ROS1* |  |
| ***CDKN2A*** | ***CDKN2B*** | *CTNNB1* | ***EGFR*** | *CDK6* |  |  |
| ***ERBB2*** | *ESR1* | *EZH2* | *FBXW7* | *EGFR* |  |  |
| ***FGFR1*** | ***FGFR2*** | *FGFR3* | *GATA3* | *ERBB2* |  |  |
| *GNA11* | *GNAQ* | *GNAS* | *HNF1A* | *FGFR1* |  |  |
| ***HRAS*** | *IDH1* | *IDH2* | *JAK2* | *FGFR2* |  |  |
| *JAK3* | ***KIT*** | ***KRAS*** | *MAP2K1* | *KIT* |  |  |
| *MAP2K2* | ***MET*** | *MLH1* | *MPL* | *KRAS* |  |  |
| ***MYC*** | ***NF1*** | *NFE2L2* | *NOTCH1* | *MET* |  |  |
| *NPM1* | ***NRAS*** | *NTRK1* | ***PDGFRA*** | *MYC* |  |  |
| ***PIK3CA*** | ***PTEN*** | *PTPN11* | ***RAF1*** | *PDGFRA* |  |  |
| *RET* | *RHEB* | *RHOA* | *RIT1* | *PIK3CA* |  |  |
| *ROS1* | *SMAD4* | *SMO* | *SRC* | *RAF1* |  |  |
| *STK11* | *TERT* | ***TP53*** | *VHL* |  |  |  |

Complete exon coverage for genes in ***bold***.

**Supplementary Table 1c.** 70 gene panel (N=27 samples).

| **POINT MUTATIONS** | | | | **AMPLIFICATIONS** | **FUSIONS** | **INDELS** |
| --- | --- | --- | --- | --- | --- | --- |
| *AKT1* | *ALK** | ***APC*** | ***AR*** | *AR* | *ALK* | *EGFR* exon 19 deletions |
| *ARAF* | ***ARID1A*** | *ATM* | ***BRAF*** | *BRAF* | *FGFR2* | *EGFR* exon 20 insertions |
| ***BRCA1*** | ***BRCA2*** | ***CCND1*** | ***CCND2*** | *CCND1* | *FGFR3* | *ERBB2* exon 19 deletions |
| ***CCNE1*** | *CDH1* | ***CDK4*** | ***CDK6*** | *CCND2* | *NTRK1* | *ERBB2* exon 20 insertions |
| ***CDKN2A*** | ***CDKN2B*** | *CTNNB1* | ***EGFR*** | *CCNE1* | *RET* |  |
| ***ERBB2*** | *ESR1* | *EZH2* | *FBXW7* | *CDK4* | *ROS1* |  |
| ***FGFR1*** | ***FGFR2**** | *FGFR3** | *GATA3* | *CDK6* |  |  |
| *GNA11* | *GNAQ* | *GNAS* | *HNF1A* | *EGFR* |  |  |
| ***HRAS*** | *IDH1* | *IDH2* | *JAK2* | *ERBB2* |  |  |
| *JAK3* | ***KIT*** | ***KRAS*** | *MAP2K1* | *FGFR1* |  |  |
| *MAP2K2* | ***MET*** | *MLH1* | *MPL* | *FGFR2* |  |  |
| ***MYC*** | ***NF1*** | *NFE2L2* | *NOTCH1* | *KIT* |  |  |
| *NPM1* | ***NRAS*** | *NTRK1** | ***PDGFRA*** | *KRAS* |  |  |
| ***PIK3CA*** | ***PTEN*** | *PTPN11* | ***RAF1*** | *MET* |  |  |
| ***RB1*** | *RET** | *RHEB* | *RHOA* | *MYC* |  |  |
| *RIT1* | *ROS1** | *SMAD4* | *SMO* | *PDGFRA* |  |  |
| *SRC* | *STK11* | *TERT* | ***TP53*** | *PIK3CA* |  |  |
| *TSC1* | *VHL* |  |  | *RAF1* |  |  |

Complete exon and partial intron coverage for genes in ***bold***. *Genes with asterisk include rearrangements. *MET* includes exon 14 skipping.

**Supplementary Table 1d.** 73 gene panel (N=64 samples).

| **POINT MUTATIONS** | | | | **AMPLIFICATIONS** | **FUSIONS** | **INDELS** | |
| --- | --- | --- | --- | --- | --- | --- | --- |
| *AKT1* | *ALK* | *APC* | *AR* | *AR* | *ALK* | *APC* | *ARID1A* |
| *ARAF* | *ARID1A* | *ATM* | *BRAF* | *BRAF* | *FGFR2* | *ATM* | *BRCA1* |
| *BRCA1* | *BRCA2* | *CCND1* | *CCND2* | *CCND1* | *FGFR3* | *BRCA2* | *CDH1* |
| *CCNE1* | *CDH1* | *CDK4* | *CDK6* | *CCNE1* | *NTRK1* | *CDKN2A* | *EGFR* |
| *CDKN2A* | *CTNNB1* | *DDR2* | *EGFR* | *CDK4* | *RET* | *GATA3* | *KIT* |
| *ERBB2* | *ESR1* | *EZH2* | *FBXW7* | *CDK6* | *ROS1* | *MET* | *MLH1* |
| *FGFR1* | *FGFR2* | *FGFR3* | *GATA3* | *EGFR* |  | *MTOR* | *NF1* |
| *GNA11* | *GNAQ* | *GNAS* | *HNF1A* | *ERBB2* |  | *PDGFRA* | *PTEN* |
| *HRAS* | *IDH1* | *IDH2* | *JAK2* | *FGFR1* |  | *RB1* | *SMAD4* |
| *JAK3* | *KIT* | *KRAS* | *MAP2K1* | *FGFR2* |  | *STK11* | *TP53* |
| *MAP2K2* | *MAPK1* | *MAPK3* | *MET* | *KIT* |  | *TSC1* | *VHL* |
| *MLH1* | *MPL* | *MTOR* | *MYC* | *KRAS* |  |  |  |
| *NF1* | *NFE2L2* | *NOTCH1* | *NPM1* | *MET* |  |  |  |
| *NRAS* | *NTRK1* | *NTRK3* | *PDGFRA* | *MYC* |  |  |  |
| *PIK3CA* | *PTEN* | *PTPN11* | *RAF1* | *PDGFRA* |  |  |  |
| *RB1* | *RET* | *RHEB* | *RHOA* | *PIK3CA* |  |  |  |
| *RIT1* | *ROS1* | *SMAD4* | *SMO* | *RAF1* |  |  |  |
| *STK11* | *TERT* | *TP53* | *TSC1* |  |  |  |  |
| *VHL* |  |  |  |  |  |  |  |

All clinically relevant exons for 73 genes are sequenced. *TERT* includes alterations in the promoter region. *MET* includes exon 14 skipping.

**Supplementary Table 2.** Genomic alteration and its potential targeted therapies with FDA-approved agents (either on/off-label) or with investigational agents in clinical trials.

| ***Genes*** | **Potential targeted therapies** | **Supplemental References** |
| --- | --- | --- |
| ***Potentially actionable with FDA approved agents (on- or off-label)*** | | |
| ***AR*** | *AR* alteration is potentially targetable with AR inhibitor enzalutamide and anti-androgens (e.g., abiraterone, bicalutamide) | S1-6 |
| ***ATM*** | *ATM* alteration is potentially targetable with PARP inhibitors (e.g., olaparib, niraparib, rucaparib). | S7-10 |
| ***BRAF*** | *BRAF* alteration can be targeted with BRAF inhibitors (e.g. dabrafenib, vemurafenib), MEK inhibitors (e.g. trametinib, cobimetinib), and multiple tyrosine kinase inhibitors (e.g., sorafenib, regorafenib) | S11-20 |
| ***BRCA1/2*** | *BRCA* alteration is targetable with platinum-based chemotherapy and PARP inhibitors (e.g., olaparib, niraparib, rucaparib). | S21-25 |
| ***CCND2*** | *CCND2* may be sensitive to CDK4/6 inhibitors (e.g., palbociclib, abemaciclib, ribociclib). | S26-28 |
| ***CDK4/6*** | *CDK6* and its functional homolog, *CDK4* mutations are theoretically targetable with CDK4/6 inhibitors (e.g., palbociclib, abemaciclib, ribociclib). | S27-30 |
| ***CDKN2A*** | *CDKN2* alterations may be sensitive to CDK4/6 inhibitors (e.g., palbociclib, abemaciclib, ribociclib). | S27, 28, 31, 32 |
| ***EGFR*** | Erlotinib, in combination with gemcitabine, has also been approved by the FDA for the treatment of locally advanced, unresectable, or metastatic pancreatic cancer. | S33, 34 |
| ***ERBB2*** | *ERBB2* alteration is targetable with Her-targeted drugs (e.g., afatinib, lapatinib, neratinib, pertuzumab, trastuzumab). | S35-40 |
| ***FBXW7*** | Although there are conflicting data, *FBXW7* aberration stabilizes the mTOR signaling which is potentially targetable with mTOR inhibitors (e.g., everolimus, temsirolimus). | S41-43 |
| ***FGFR1*** | *FGFR1* alteration is targetable with multi-kinase inhibitors (e.g., pazopanib, ponatinib, regorafenib, nintedanib, lenvatinib). | S13, 14, 44-48 |
| ***GNAS*** | *GNAS* alteration is potentially targetable with MEK inhibitors (e.g., trametinib, cobimetinib). | S18, 19, 49-51 |
| ***IDH1*** | *IDH1* alteration is targetable with IDH1 inhibitors (e.g., ivosidenib). | S52-54 |
| ***KRAS*** | *KRAS* alteration is potentially targetable with MEK inhibitors (e.g., trametinib, cobimetinib). | S50, 55-63 |
| ***MET*** | *MET* alteration is targetable with multi-kinase inhibitors (e.g., cabozantinib, crizotinib). | S64-68 |
| ***MPL*** | *MPL* alterations is associated with increased Jak/Stat signaling. Thus, it may be targetable with JAK inhibitors (e.g., ruxolitinib). | S69-72 |
| ***MTOR*** | *MTOR* alteration is potentially targetable with mTOR inhibitors (e.g., everolimus, temsirolimus). | S73-79 |
| ***NF1*** | *NF1* alteration is associated with activation of RAS and downstream pathways. Thus, it may be targetable with MEK inhibitors (e.g., trametinib, cobimetinib). | S18, 19, 80, 81 |
| ***PIK3CA*** | *PIK3CA* alteration is targetable with mTOR inhibitors (e.g., everolimus, temsirolimus). | S78, 79, 82-86 |
| ***PTEN*** | *PTEN* alteration is targetable with mTOR inhibitors (e.g., everolimus, temsirolimus). | S78, 79, 87 |
| ***TP53*** | Retrospective data suggest patients with *TP53* mutation had longer progression-free survival with bevacizumab containing regimen when compared to non-bevacizumab containing regimen (median 11.0 vs. 4.0 months, p<0.0001). *TP53* alteration status was also predictive of longer progression-free survival among sarcoma patients treated with pazopanib (multi-kinase inhibitor including VEGF) (hazard ratio: 0.38, p = 0.036). Interestingly, multiple regression analysis of transcriptomic data revealed *TP53* mutations are associated with higher VEGFA expression (p = 0.0006) suggesting the TP53 as a marker to predict bevacizumab response. | S88-91 |
| ***Potentially actionable with investigational agents*** | | |
| ***MYC*** | *MYC* alteration is potentially targetable with BET inhibitors (e.g., GSK-525762). | S92-94 |

**Supplementary Table 3.** Assessment of genomic uniqueness in ctDNA among patients with pancreatic ductal adenocarcinoma.

| **Altered gene** | **Study ID** | **Characterized alterations** |
| --- | --- | --- |
| *TP53*, *KRAS, and CDKN2A* | 7 | *TP53* E56*, *KRAS* G12V, *CDKN2A* G23V |
|  | 51 | *TP53* C124*, *KRAS* G12R, *CDKN2A* R103fs |
| *TP53* and *KRAS* | 3 | *TP53* R175H, *KRAS* G12D |
|  | 14 | *TP53* Splice Site SNV, *KRAS* G12D |
|  | 31 | *TP53* C275F, *KRAS* G12V |
|  | 74 | *TP53* R248W, *KRAS* Q61H |
|  | 76 | *TP53* R196*, *KRAS* Q61H, |
|  | 87 | *TP53* R248W, *TP53* R213*, *TP53* P222L, *KRAS* G12C |
|  | 95 | *TP53* R273C, *KRAS* G12D |
|  | 96 | *TP53* S127F, *TP53* A189T, *KRAS* G12D |
|  | 101 | *TP53* C135G, *KRAS* G12V |
|  | 108 | *TP53* R158fs, *KRAS* G12V |
| *TP53* | 24 | *TP53* V216M |
|  | 28 | *TP53* Q100R |
|  | 29 | *TP53* H214R |
|  | 40 | *TP53* Y220C |
|  | 41 | *TP53* E285K, *TP53* E221K |
|  | 42 | *TP53* I195T |
|  | 53 | *TP53* L93M |
|  | 55 | *TP53* R213* |
|  | 59 | *TP53* R196P |
|  | 68 | *TP53* R196* |
|  | 71 | *TP53* R175H |
|  | 86 | *TP53* R273H |
|  | 93 | *TP53* V218E |
|  | 102 | *TP53* V216M |
|  | 106 | *TP53* Q100fs |
| *KRAS* | 8 | *KRAS* G12R |
|  | 43 | *KRAS* Q61H |
|  | 50 | *KRAS* G12D |
|  | 60 | *KRAS* G12V, *KRAS* Amplification |
|  | 64 | *KRAS* G12V |
|  | 70 | *KRAS* G12V |
|  | 85 | *KRAS* G12R |
|  | 88 | *KRAS* G12V, *KRAS* Amplification |

**Supplementary Table 4**. Complete list of ctDNA alterations found in patients with pancreatic ductal adenocarcinoma (N=112).

| **ID** | **Characterized alteration in ctDNA** | **Disease status at the time of ctDNA analysis**  **(all treatment regimens prior to ctDNA)** |
| --- | --- | --- |
| 1 | no characterized alteration detectable | Surgically resectable *blood obtained after surgery |
| 2 | no characterized alteration detectable | Metastatic, locally advanced, or recurrent disease  (capecitabine; gemcitabine + abraxane; FOLFIRINOX) |
| 3 | *KRAS* G12D, *TP53* R175H | Metastatic, locally advanced, or recurrent disease (no prior chemotherapy) |
| 4 | no characterized alteration detectable | Metastatic, locally advanced, or recurrent disease  (FOLFIRINOX; gemcitabine + abraxane) |
| 5 | no characterized alteration detectable | Surgically resectable *blood obtained before surgery |
| 6 | *KRAS* G12D, *SMAD4* R361C, *TP53* H214P, *GNAS* R201C, *ATM* R3047Q | Metastatic, locally advanced, or recurrent disease (no prior chemotherapy) |
| 7 | *CDKN2A* G23V, *KRAS* G12V, *TP53* E56* | Metastatic, locally advanced, or recurrent disease  (gemcitabine; capecitabine) |
| 8 | *KRAS* G12R | Metastatic, locally advanced, or recurrent disease (no prior chemotherapy) |
| 9 | no characterized alteration detectable | Surgically resectable *blood obtained before surgery |
| 10 | no characterized alteration detectable | Surgically resectable *blood obtained after surgery |
| 11 | no characterized alteration detectable | Metastatic, locally advanced, or recurrent disease (no prior chemotherapy) |
| 12 | *TERT* Promoter SNV | Metastatic, locally advanced, or recurrent disease  (gemcitabine + abraxane) |
| 13 | no characterized alteration detectable | Metastatic, locally advanced, or recurrent disease (no prior chemotherapy) |
| 14 | *TP53* Splice Site SNV, *KRAS* G12D | Metastatic, locally advanced, or recurrent disease (no prior chemotherapy) |
| 15 | *PIK3CA* E545K | Metastatic, locally advanced, or recurrent disease (no prior chemotherapy) |
| 16 | *GNAS* R201H, *KRAS* G12V, *TP53* L265P | Metastatic, locally advanced, or recurrent disease (no prior chemotherapy) |
| 17 | no characterized alteration detectable | Surgically resectable *blood obtained before surgery |
| 18 | *AR* R775H | Metastatic, locally advanced, or recurrent disease  (FOLFIRINOX) |
| 19 | *KRAS* G12V, *TP53* R306*, *PIK3CA* E545K, *NF1* K1444E | Metastatic, locally advanced, or recurrent disease (no prior chemotherapy) |
| 20 | no characterized alteration detectable | Surgically resectable *blood obtained after surgery |
| 21 | no characterized alteration detectable | Metastatic, locally advanced, or recurrent disease (no prior chemotherapy) |
| 22 | *KRAS* G12D, *TP53* C176F, *PTEN* R13Q | Metastatic, locally advanced, or recurrent disease (no prior chemotherapy) |
| 23 | no characterized alteration detectable | Metastatic, locally advanced, or recurrent disease (no prior chemotherapy) |
| 24 | *TP53* V216M | Metastatic, locally advanced, or recurrent disease (no prior chemotherapy) |
| 25 | no characterized alteration detectable | Metastatic, locally advanced, or recurrent disease (no prior chemotherapy) |
| 26 | *MPL* W515L, *TP53* C141Y | Surgically resectable *blood obtained before surgery |
| 27 | *NF1* I679fs, *GNAS* R201C | Metastatic, locally advanced, or recurrent disease  (gemcitabine + abraxane) |
| 28 | *TP53* Q100R | Metastatic, locally advanced, or recurrent disease (no prior chemotherapy) |
| 29 | *TP53* H214R | Surgically resectable *blood obtained after surgery |
| 30 | *CCND2* amplification | Surgically resectable *blood obtained before surgery |
| 31 | *TP53* C275F, *KRAS* G12V | Metastatic, locally advanced, or recurrent disease (no prior chemotherapy) |
| 32 | no characterized alteration detectable | Surgically resectable *blood obtained after surgery |
| 33 | *TP53* R213L, *FBXW7* R385H | Metastatic, locally advanced, or recurrent disease  (5FU+ abraxane + oxaliplatin + bevacizuamb; gemcitabine + abraxane; 5FU) |
| 34 | *KRAS* G12V, *MYC* amplification | Metastatic, locally advanced, or recurrent disease  (gemcitabine) |
| 35 | *TP53* S215R, *MYC* amplification | Metastatic, locally advanced, or recurrent disease (no prior chemotherapy) |
| 36 | no characterized alteration detectable | Metastatic, locally advanced, or recurrent disease (no prior chemotherapy) |
| 37 | no characterized alteration detectable | Metastatic, locally advanced, or recurrent disease  (FOLFIRINOX; gemcitabine + abraxane) |
| 38 | *RB1* E282*, *TP53* R273C, *KRAS* G12D | Surgically resectable *blood obtained before surgery |
| 39 | *TP53* R282W, *KRAS* G12R, *CDKN2A* W15*, *ERBB2* amplification, *CDK4* amplification, *KRAS* amplification | Metastatic, locally advanced, or recurrent disease (no prior chemotherapy) |
| 40 | *TP53* Y220C | Metastatic, locally advanced, or recurrent disease  (gemcitabine + carboplatin) |
| 41 | *TP53* E285K, *TP53* E221K | Metastatic, locally advanced, or recurrent disease (no prior chemotherapy) |
| 42 | *TP53* I195T | Metastatic, locally advanced, or recurrent disease (no prior chemotherapy) |
| 43 | *KRAS* Q61H | Metastatic, locally advanced, or recurrent disease  (FOLFIRINOX; gemcitabine + abraxane) |
| 44 | no characterized alteration detectable | Metastatic, locally advanced, or recurrent disease  (capecitabine) |
| 45 | no characterized alteration detectable | Metastatic, locally advanced, or recurrent disease (no prior chemotherapy) |
| 46 | no characterized alteration detectable | Metastatic, locally advanced, or recurrent disease (no prior chemotherapy) |
| 47 | *CDKN2A* R80*, *KRAS* G12D | Metastatic, locally advanced, or recurrent disease  (abraxane + palbociclib; gemcitabine + erlotinib; palbociclib + trametinib + anakinra; palbociclib + trametinib + afatinib) |
| 48 | no characterized alteration detectable | Metastatic, locally advanced, or recurrent disease  (capecitabine) |
| 49 | *KRAS* G12R, *SMAD4* R361G, *CDK6* amplification, *EGFR* amplification | Metastatic, locally advanced, or recurrent disease  (gemcitabine + abraxane) |
| 50 | *KRAS* G12D | Metastatic, locally advanced, or recurrent disease  (gemcitabine + capecitabine; abraxane) |
| 51 | *KRAS* G12R, *TP53* C124*, *CDKN2A* R103fs | Metastatic, locally advanced, or recurrent disease  (FOLFIRINOX) |
| 52 | no characterized alteration detectable | Metastatic, locally advanced, or recurrent disease (no prior chemotherapy) |
| 53 | *TP53* L93M | Metastatic, locally advanced, or recurrent disease (no prior chemotherapy) |
| 54 | *TP53* V172F, *CCNE1* amplification, *BRAF* amplification, *EGFR* amplification, *CDK6* amplification | Metastatic, locally advanced, or recurrent disease  (gemcitabine) |
| 55 | *TP53* R213* | Metastatic, locally advanced, or recurrent disease (no prior chemotherapy) |
| 56 | no characterized alteration detectable | Metastatic, locally advanced, or recurrent disease  (FOLFIRINOX) |
| 57 | no characterized alteration detectable | Metastatic, locally advanced, or recurrent disease  (gemcitabine) |
| 58 | *KRAS* G12L, *FGFR1* amplification | Metastatic, locally advanced, or recurrent disease (no prior chemotherapy) |
| 59 | *TP53* R196P | Surgically resectable *blood obtained before surgery |
| 60 | *KRAS* G12V, *KRAS* amplification | Metastatic, locally advanced, or recurrent disease (no prior chemotherapy) |
| 61 | *KRAS* G12R, *TP53* P223L, *MYC* amplification | Metastatic, locally advanced, or recurrent disease (no prior chemotherapy) |
| 62 | no characterized alteration detectable | Metastatic, locally advanced, or recurrent disease  (gemcitabine + abraxane; capecitabine) |
| 63 | *GNAS* R201H | Metastatic, locally advanced, or recurrent disease  (FOLFIRINOX; gemcitabine + abraxane) |
| 64 | *KRAS* G12V | Metastatic, locally advanced, or recurrent disease (no prior chemotherapy) |
| 65 | *KRAS* G12D, *TP53* G105V, *GNAS* R201C, *KRAS* Amplification, *MYC* Amplification, *MET* Amplification | Metastatic, locally advanced, or recurrent disease (no prior chemotherapy) |
| 66 | no characterized alteration detectable | Surgically resectable *blood obtained before surgery |
| 67 | *IDH1* R132C | Metastatic, locally advanced, or recurrent disease  (gemcitabine + abraxane) |
| 68 | *TP53* R196* | Metastatic, locally advanced, or recurrent disease  (capecitabine) |
| 69 | no characterized alteration detectable | Metastatic, locally advanced, or recurrent disease  (gemcitabine + abraxane; capecitabine + irinotecan) |
| 70 | *KRAS* G12V | Metastatic, locally advanced, or recurrent disease (no prior chemotherapy) |
| 71 | *TP53* R175H | Metastatic, locally advanced, or recurrent disease  (gemcitabine + abraxane; FOLFOX) |
| 72 | *KRAS* G12R, *TP53* Y220C, *TP53* K132E, *PIK3CA* amplification, *CCND2* amplification, *CCNE1* amplification | Metastatic, locally advanced, or recurrent disease  (capecitabine + oxaliplatin; capecitabine) |
| 73 | *KRAS* G12V, *TP53* C176W, *FBXW7* R465C, *FGFR1* amplification, *BRAF* amplification | Metastatic, locally advanced, or recurrent disease (no prior chemotherapy) |
| 74 | *TP53* R248W, *KRAS* Q61H | Metastatic, locally advanced, or recurrent disease (no prior chemotherapy) |
| 75 | no characterized alteration detectable | Metastatic, locally advanced, or recurrent disease  (FOLFIRINOX) |
| 76 | *KRAS* Q61H, *TP53* R196* | Metastatic, locally advanced, or recurrent disease (no prior chemotherapy) |
| 77 | *KRAS* G12D, *MYC* Amplification, *CCNE1* Amplification, *PIK3CA* Amplification, *KRAS* Amplification | Metastatic, locally advanced, or recurrent disease (no prior chemotherapy) |
| 78 | no characterized alteration detectable | Metastatic, locally advanced, or recurrent disease  (FOLFIRINOX) |
| 79 | *KRAS* Q61R, SMAD4 D537Y, *TP53* Y163C, *KRAS* Amplification | Metastatic, locally advanced, or recurrent disease (no prior chemotherapy) |
| 80 | *KRAS* G12V, *TP53* Y220H, *CCNE1* amplification, *PIK3CA* R401* | Metastatic, locally advanced, or recurrent disease  (gemcitabine + abraxane) |
| 81 | no characterized alteration detectable | Surgically resectable *blood obtained after surgery |
| 82 | no characterized alteration detectable | Metastatic, locally advanced, or recurrent disease (no prior chemotherapy) |
| 83 | *KRAS* G12R, *TP53* R282W, *KRAS* Amplification, MYC Amplification, *PIK3CA* Amplification | Metastatic, locally advanced, or recurrent disease (no prior chemotherapy) |
| 84 | *BRAF* G469V | Metastatic, locally advanced, or recurrent disease  (gemcitabine + abraxane + momelotinib; FOLFIRINOX; cediranib + olaparib) |
| 85 | *KRAS* G12R | Metastatic, locally advanced, or recurrent disease  (gemcitabine + abraxane) |
| 86 | *TP53* R273H | Metastatic, locally advanced, or recurrent disease (no prior chemotherapy) |
| 87 | *KRAS* G12C, *TP53* R248W, *TP53* R213*, *TP53* P222L | Metastatic, locally advanced, or recurrent disease (no prior chemotherapy) |
| 88 | *KRAS* G12V, *KRAS* Amplification | Metastatic, locally advanced, or recurrent disease (no prior chemotherapy) |
| 89 | *KRAS* G12D, KRAS amplification, *TP53* C242fs, *TP53* H168fs, *BRCA2* T3033fs, *IDH1* R132H | Metastatic, locally advanced, or recurrent disease  (FOLFIRINOX; gemcitabine + abraxane) |
| 90 | *KRAS* G12D, *TP53* R273P, *NF1* N1652K | Metastatic, locally advanced, or recurrent disease  (FOLFIRINOX; capecitabine) |
| 91 | *ATM* R337H, *KRAS* G12D | Metastatic, locally advanced, or recurrent disease (no prior chemotherapy) |
| 92 | *BRCA1* Splice Site SNV, *KRAS* G12V, *TP53* S149fs | Metastatic, locally advanced, or recurrent disease (no prior chemotherapy) |
| 93 | *TP53* V218E | Surgically resectable *blood obtained after surgery |
| 94 | no characterized alteration detectable | Metastatic, locally advanced, or recurrent disease (no prior chemotherapy) |
| 95 | *TP53* R273C, *KRAS* G12D | Metastatic, locally advanced, or recurrent disease  (gemcitabine + abraxane; FOLFIRINOX) |
| 96 | *TP53* S127F, *KRAS* G12D, *TP53* A189T | Metastatic, locally advanced, or recurrent disease  (gemcitabine) |
| 97 | *GNAS* R201H, *CDKN2A* E27*, *TP53* splice Site SNV, *KRAS* G12D | Metastatic, locally advanced, or recurrent disease  (FOLFIRINOX) |
| 98 | *KRAS* G12D, *GNAS* R201H, *ATM* Splice Site SNV | Metastatic, locally advanced, or recurrent disease  (capecitabine; gemcitabine + abraxane; FOLFOX; 5FU + irinotecan) |
| 99 | *KRAS* G12D, *TP53* P177_C182del, *SMAD4* S232fs, *CDKN2A* H83Y, *NF1* Y489C, *TP53* R181fs | Metastatic, locally advanced, or recurrent disease (no prior chemotherapy) |
| 100 | no characterized alteration detectable | Metastatic, locally advanced, or recurrent disease (no prior chemotherapy) |
| 101 | *KRAS* G12V, *TP53* C135G | Metastatic, locally advanced, or recurrent disease  (capecitabine + oxaliplatin; gemcitabine + abraxane) |
| 102 | *TP53* V216M | Surgically resectable *blood obtained before surgery |
| 103 | *ARID1A* W588fs | Surgically resectable *blood obtained after surgery |
| 104 | *KRAS* G12V, *CDKN2A* R80*, *CDK6* amplification, *SMAD4* V341Fs, *TP53* Q331H1 | Metastatic, locally advanced, or recurrent disease (no prior chemotherapy) |
| 105 | *NF1* Splice Site SNV, *NF1* I2078fs | Metastatic, locally advanced, or recurrent disease (no prior chemotherapy) |
| 106 | *TP53* Q100fs | Metastatic, locally advanced, or recurrent disease  (gemcitabine + abraxane; FOLFIRINOX) |
| 107 | *CDKN2A* Splice Site SNV, *KRAS* G12D, *TP53* Y236C, *SMAD4* H305fs | Metastatic, locally advanced, or recurrent disease (no prior chemotherapy) |
| 108 | *TP53* R158fs, *KRAS* G12V | Metastatic, locally advanced, or recurrent disease (no prior chemotherapy) |
| 109 | no characterized alteration detectable | Surgically resectable *blood obtained before surgery |
| 110 | *ATM* R3008H, *CDK6* amplification, *KRAS* G12R | Metastatic, locally advanced, or recurrent disease (no prior chemotherapy) |
| 111 | *GNAS* R201C, *NF1* D1976fs, *KRAS* G12D, *EGFR* G1022S, *MTOR* D258fs | Metastatic, locally advanced, or recurrent disease (no prior chemotherapy) |
| 112 | no characterized alteration detectable | Metastatic, locally advanced, or recurrent disease (no prior chemotherapy) |

**Supplementary Table 5**. Comparisons of ctDNA parameters between refractory and treatment naïve cases (N=94 with advanced pancreatic ductal adenocarcinoma).

| ***Parameters*** | ***Patients with ≥1 chemotherapy regimen prior to blood draw for ctDNA***  ***(N=40)*** | ***Patients with no chemotherapy regimen prior to blood draw for ctDNA***  ***(N=54)*** | ***P-*value** |
| --- | --- | --- | --- |
| **Number of characterized alterations**  Median (range) | 1 (0-6) | 2 (0-6) | 0.27 |
| **≥1 characterized ctDNA alteration detected**  *TP53* alteration  *KRAS* alteration | 29 (73%)  16 (40.0%)  17 (43%) | 41 (76%)  29 (54%)  31 (57%) | 0.81  0.22  0.21 |
| **Maximum %ctDNA per patient**  Median (range) (%) | 0.3 (0.0-62.5) | 0.6 (0.0-64.6) | 0.29 |
| **Total %ctDNA per patient**  Median (range) (%) | 0.5 (0.0-92.5) | 0.9 (0.0--86.6) | 0.34 |

**Supplementary Table 6**. Patients who had full concordant results between ctDNA and tissue DNA analyses.

| **ID** | **All characterized alterations in ctDNA** | **Disease status and intervention at the time of ctDNA analysis** | **All characterized alterations in tissue DNA** | **Disease status and intervention at the time of ctDNA analysis** | **Tissue biopsy site** | **Time between blood draw and tissue biopsy** |
| --- | --- | --- | --- | --- | --- | --- |
| 51 | *CDKN2A* R103fs, *KRAS* G12R, *TP53* C124* | Metastatic    Blood obtained after disease progression from 1st line therapy | *CDKN2A* p16INK4a R103fs*40 and p14ARF A117fs*52, *KRAS* G12R, *TP53* C124* | Metastatic    Tissue obtained before 1st line therapy | Metastatic site (liver) | 5.9 month |
| 61 | *KRAS* G12R, *MYC* Amplification, *TP53* P223L | Metastatic    Blood obtained before 1st line therapy | *KRAS* G12R, *MYC* amplification, TP53 P223fs*24 | Metastatic    Tissue obtained before 1st line therapy | Metastatic site (liver) | 0.4 months |
| 79 | *KRAS* Amplification, *KRAS* G61R, *SMAD4* D537Y, *TP53* Y163C | Metastatic    Blood obtained before 1st line therapy | *KRAS* Q61R, *SMAD4* D537Y, *TP53* Y163C | Metastatic    Tissue obtained before 1st line therapy | Metastatic site (liver) | 1.0 months |
| 97 | *CDKN2A* E27*, *GNAS* R201H, *KRAS* G12D, *TP53* splice Site SNV | Metastatic    Blood obtained before 2nd line therapy | *CDKN2A* p16INK4a E27*, *GNAS* R201H, *KRAS* G12D, *TP53* splice site 919+2T>G | Metastatic    Tissue obtained during 1st line therapy | Metastatic site (liver) | 4.6 months |

**Supplementary Figure 1**. Consort diagram for tumors assessed by next-generation sequencing of DNA.


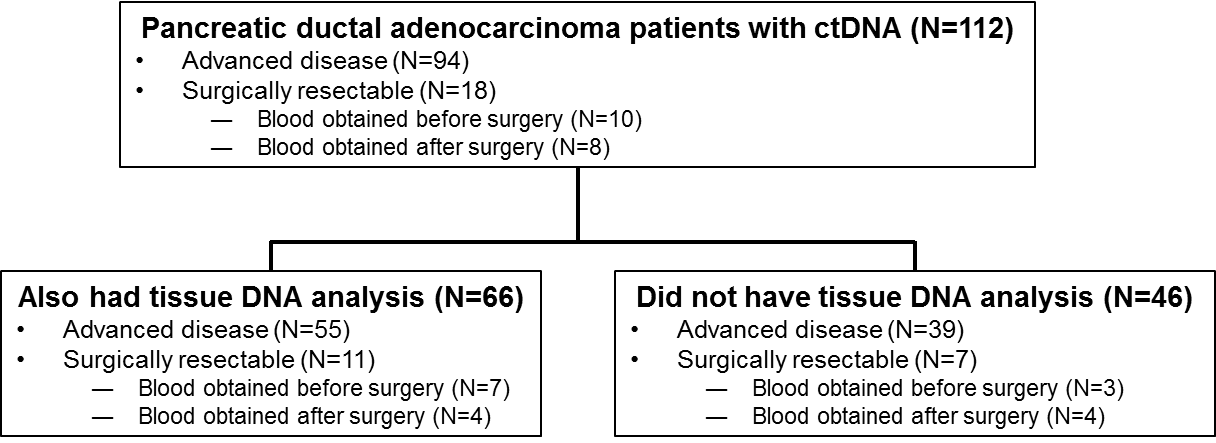


**Supplementary Figure 2**. Kaplan-Meier curve for overall survival from advanced disease depending on %ctDNA dichotomized at median (for total %ctDNA for all alterations) among patients with advanced PDAC (N=94).


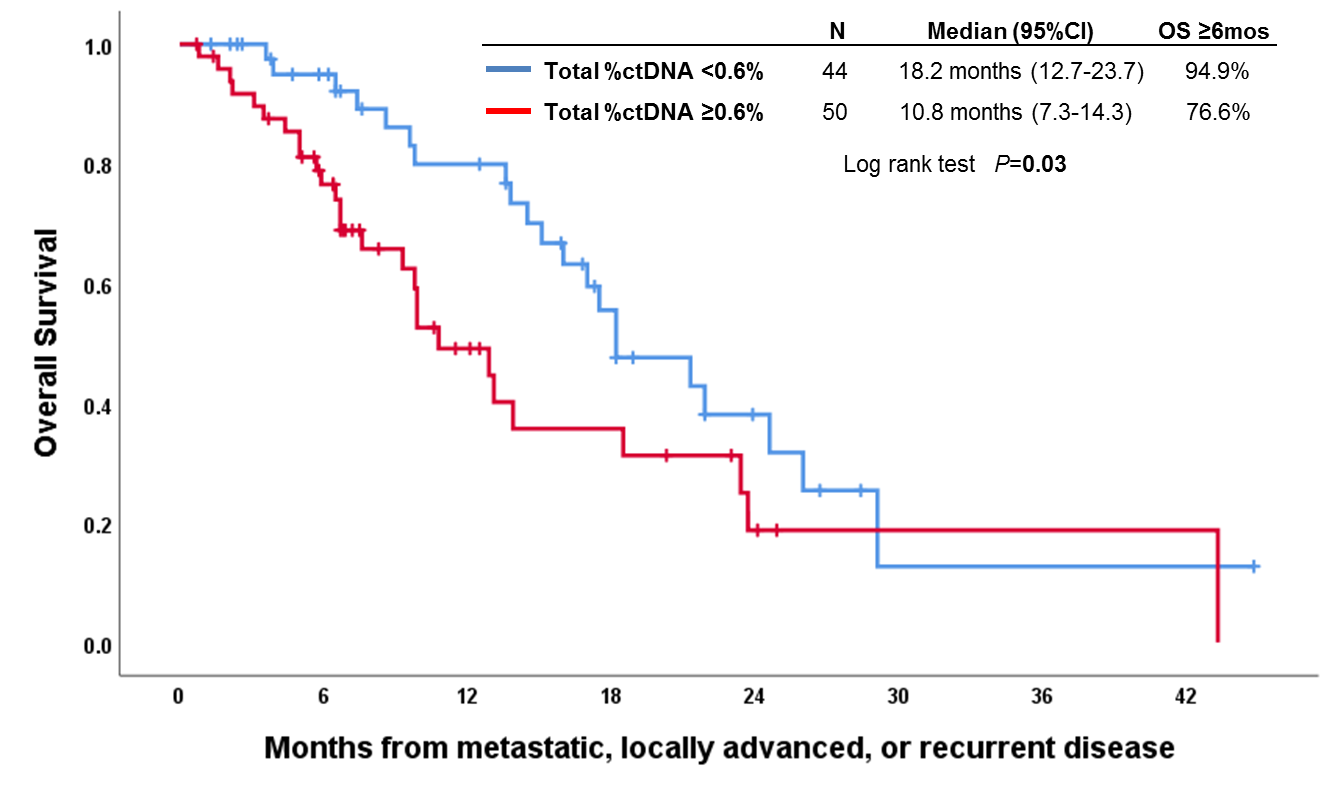


**Supplementary Figure 3**. Kaplan-Meier curve for overall survival from ctDNA analysis stratified by ctDNA sequencing panel.

**Supplementary Figure 3a**. 73-gene panel [N=52].


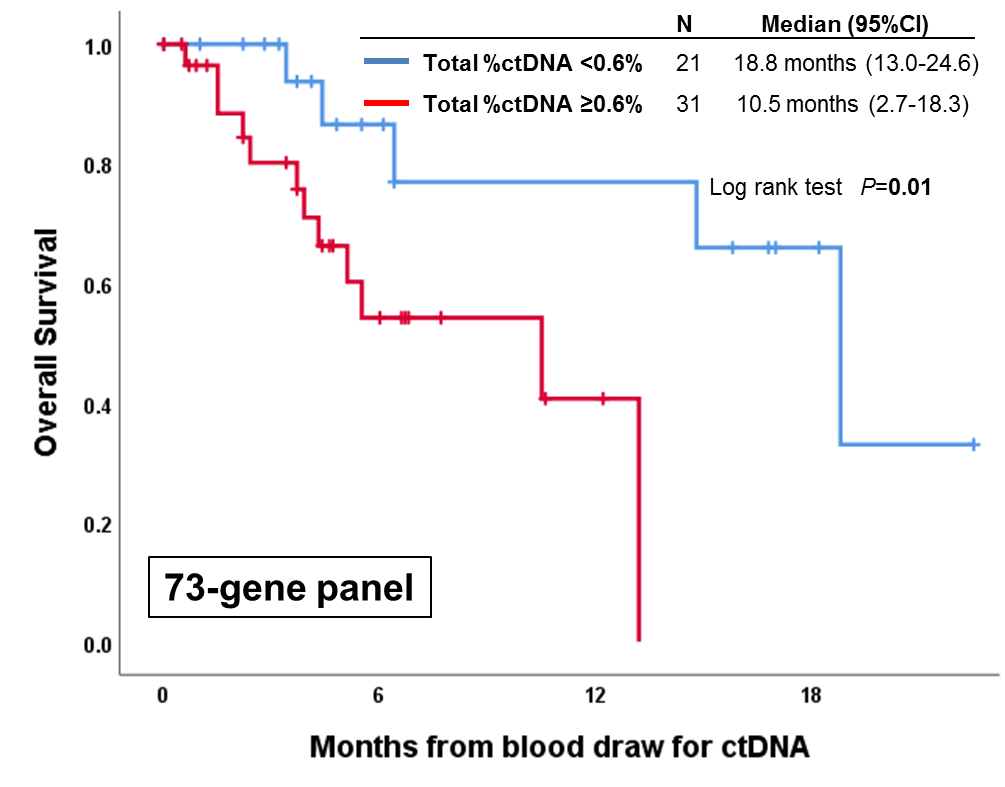


**Supplementary Figure 3b**. 54-70-gene panels [N=42].

**
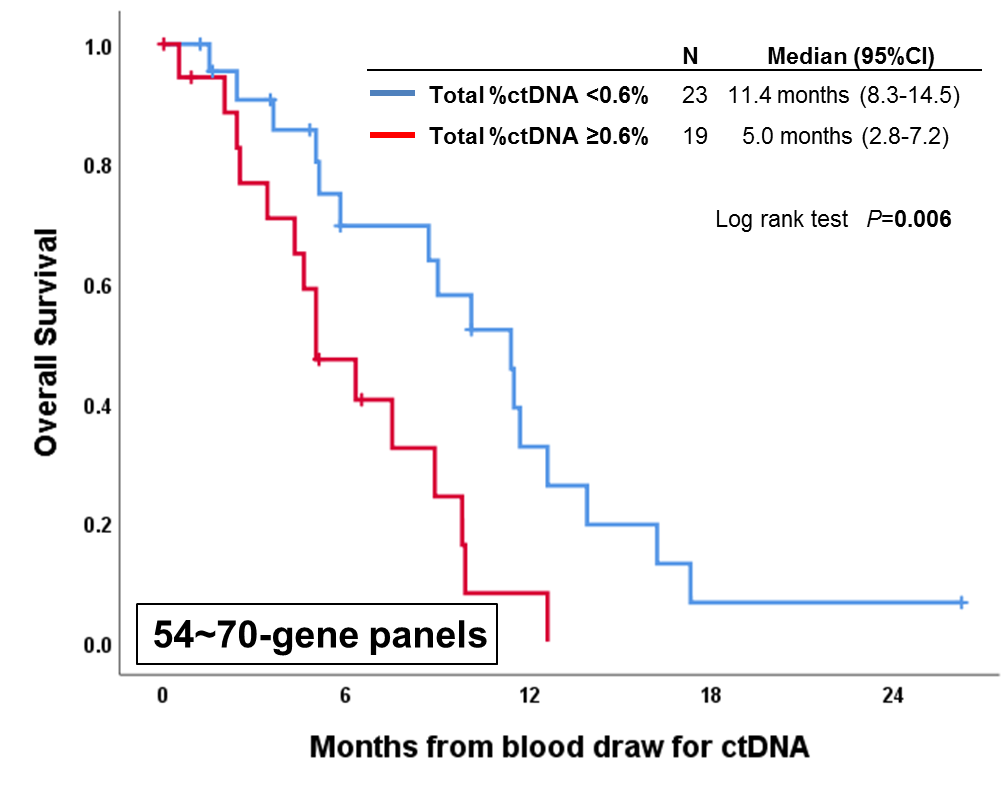
**

**SUPPLEMENTAL REFERENCES**

1. Helsen C, Van den Broeck T, Voet A, et al. Androgen receptor antagonists for prostate cancer therapy. Endocr Relat Cancer 2014;21:T105-18.

2. Gelman IH. Androgen receptor activation in castration-recurrent prostate cancer: the role of Src-family and Ack1 tyrosine kinases. Int J Biol Sci 2014;10:620-6.

3. Sharifi N. Mechanisms of androgen receptor activation in castration-resistant prostate cancer. Endocrinology 2013;154:4010-7.

4. Sartor O. Combination therapy: Abiraterone prolongs survival in metastatic prostate cancer. Nat Rev Clin Oncol 2011;8:515-6.

5. Scher HI, Fizazi K, Saad F, et al. Increased survival with enzalutamide in prostate cancer after chemotherapy. N Engl J Med 2012;367:1187-97.

6. Akaza H, Hinotsu S, Usami M, et al. Combined androgen blockade with bicalutamide for advanced prostate cancer: long-term follow-up of a phase 3, double-blind, randomized study for survival. Cancer 2009;115:3437-45.

7. Peng G, Lin SY. Exploiting the homologous recombination DNA repair network for targeted cancer therapy. World J Clin Oncol 2011;2:73-9.

8. Weston VJ, Oldreive CE, Skowronska A, et al. The PARP inhibitor olaparib induces significant killing of ATM-deficient lymphoid tumor cells in vitro and in vivo. Blood 2010;116:4578-87.

9. Riabinska A, Daheim M, Herter-Sprie GS, et al. Therapeutic targeting of a robust non-oncogene addiction to PRKDC in ATM-defective tumors. Sci Transl Med 2013;5:189ra78.

10. Michels J, Vitale I, Saparbaev M, et al. Predictive biomarkers for cancer therapy with PARP inhibitors. Oncogene 2014;33:3894-907.

11. Aprile G, Macerelli M, Giuliani F. Regorafenib for gastrointestinal malignancies : from preclinical data to clinical results of a novel multi-target inhibitor. BioDrugs 2013;27:213-24.

12. Wilhelm SM, Dumas J, Adnane L, et al. Regorafenib (BAY 73-4506): a new oral multikinase inhibitor of angiogenic, stromal and oncogenic receptor tyrosine kinases with potent preclinical antitumor activity. Int J Cancer 2011;129:245-55.

13. Grothey A, Van Cutsem E, Sobrero A, et al. Regorafenib monotherapy for previously treated metastatic colorectal cancer (CORRECT): an international, multicentre, randomised, placebo-controlled, phase 3 trial. Lancet 2013;381:303-12.

14. Demetri GD, Reichardt P, Kang YK, et al. Efficacy and safety of regorafenib for advanced gastrointestinal stromal tumours after failure of imatinib and sunitinib (GRID): an international, multicentre, randomised, placebo-controlled, phase 3 trial. Lancet 2013;381:295-302.

15. Bruix J, Qin S, Merle P, et al. Regorafenib for patients with hepatocellular carcinoma who progressed on sorafenib treatment (RESORCE): a randomised, double-blind, placebo-controlled, phase 3 trial. Lancet 2017;389:56-66.

16. Flaherty KT, Puzanov I, Kim KB, et al. Inhibition of mutated, activated BRAF in metastatic melanoma. N Engl J Med 2010;363:809-19.

17. Flaherty KT, Infante JR, Daud A, et al. Combined BRAF and MEK inhibition in melanoma with BRAF V600 mutations. N Engl J Med 2012;367:1694-703.

18. Flaherty KT, Robert C, Hersey P, et al. Improved survival with MEK inhibition in BRAF-mutated melanoma. N Engl J Med 2012;367:107-14.

19. Larkin J, Ascierto PA, Dreno B, et al. Combined vemurafenib and cobimetinib in BRAF-mutated melanoma. N Engl J Med 2014;371:1867-76.

20. Dummer R, Ascierto PA, Gogas HJ, et al. Encorafenib plus binimetinib versus vemurafenib or encorafenib in patients with BRAF-mutant melanoma (COLUMBUS): a multicentre, open-label, randomised phase 3 trial. Lancet Oncol 2018;19:603-615.

21. Hollis RL, Churchman M, Gourley C. Distinct implications of different BRCA mutations: efficacy of cytotoxic chemotherapy, PARP inhibition and clinical outcome in ovarian cancer. Onco Targets Ther 2017;10:2539-2551.

22. Kim G, Ison G, McKee AE, et al. FDA Approval Summary: Olaparib Monotherapy in Patients with Deleterious Germline BRCA-Mutated Advanced Ovarian Cancer Treated with Three or More Lines of Chemotherapy. Clin Cancer Res 2015;21:4257-61.

23. Swisher EM, Lin KK, Oza AM, et al. Rucaparib in relapsed, platinum-sensitive high-grade ovarian carcinoma (ARIEL2 Part 1): an international, multicentre, open-label, phase 2 trial. Lancet Oncol 2017;18:75-87.

24. Mirza MR, Monk BJ, Herrstedt J, et al. Niraparib Maintenance Therapy in Platinum-Sensitive, Recurrent Ovarian Cancer. N Engl J Med 2016;375:2154-2164.

25. Tutt A, Robson M, Garber JE, et al. Oral poly(ADP-ribose) polymerase inhibitor olaparib in patients with BRCA1 or BRCA2 mutations and advanced breast cancer: a proof-of-concept trial. Lancet 2010;376:235-44.

26. Gong X, Litchfield LM, Webster Y, et al. Genomic Aberrations that Activate D-type Cyclins Are Associated with Enhanced Sensitivity to the CDK4 and CDK6 Inhibitor Abemaciclib. Cancer Cell 2017;32:761-776.e6.

27. DeMichele A, Clark AS, Tan KS, et al. CDK 4/6 inhibitor palbociclib (PD0332991) in Rb+ advanced breast cancer: phase II activity, safety, and predictive biomarker assessment. Clin Cancer Res 2015;21:995-1001.

28. Sherr CJ, Beach D, Shapiro GI. Targeting CDK4 and CDK6: From Discovery to Therapy. Cancer Discov 2016;6:353-67.

29. Lapenna S, Giordano A. Cell cycle kinases as therapeutic targets for cancer. Nat Rev Drug Discov 2009;8:547-66.

30. Malumbres M, Pevarello P, Barbacid M, et al. CDK inhibitors in cancer therapy: what is next? Trends Pharmacol Sci 2008;29:16-21.

31. Zhang Y, Xiong Y, Yarbrough WG. ARF promotes MDM2 degradation and stabilizes p53: ARF-INK4a locus deletion impairs both the Rb and p53 tumor suppression pathways. Cell 1998;92:725-34.

32. Stone S, Jiang P, Dayananth P, et al. Complex structure and regulation of the P16 (MTS1) locus. Cancer Res 1995;55:2988-94.

33. Moore MJ, Goldstein D, Hamm J, et al. Erlotinib plus gemcitabine compared with gemcitabine alone in patients with advanced pancreatic cancer: a phase III trial of the National Cancer Institute of Canada Clinical Trials Group. J Clin Oncol 2007;25:1960-6.

34. Senderowicz AM, Johnson JR, Sridhara R, et al. Erlotinib/gemcitabine for first-line treatment of locally advanced or metastatic adenocarcinoma of the pancreas. Oncology (Williston Park) 2007;21:1696-706; discussion 1706-9, 1712, 1715.

35. Bang YJ, Van Cutsem E, Feyereislova A, et al. Trastuzumab in combination with chemotherapy versus chemotherapy alone for treatment of HER2-positive advanced gastric or gastro-oesophageal junction cancer (ToGA): a phase 3, open-label, randomised controlled trial. Lancet 2010;376:687-97.

36. Jones KL, Buzdar AU. Evolving novel anti-HER2 strategies. Lancet Oncol 2009;10:1179-87.

37. Baselga J, Cortes J, Kim SB, et al. Pertuzumab plus trastuzumab plus docetaxel for metastatic breast cancer. N Engl J Med 2012;366:109-19.

38. Verma S, Miles D, Gianni L, et al. Trastuzumab emtansine for HER2-positive advanced breast cancer. N Engl J Med 2012;367:1783-91.

39. Gelmon KA, Boyle FM, Kaufman B, et al. Lapatinib or Trastuzumab Plus Taxane Therapy for Human Epidermal Growth Factor Receptor 2-Positive Advanced Breast Cancer: Final Results of NCIC CTG MA.31. J Clin Oncol 2015;33:1574-83.

40. Modjtahedi H, Cho BC, Michel MC, et al. A comprehensive review of the preclinical efficacy profile of the ErbB family blocker afatinib in cancer. Naunyn Schmiedebergs Arch Pharmacol 2014;387:505-21.

41. Mao JH, Kim IJ, Wu D, et al. FBXW7 targets mTOR for degradation and cooperates with PTEN in tumor suppression. Science 2008;321:1499-502.

42. Jardim DL, Wheler JJ, Hess K, et al. FBXW7 mutations in patients with advanced cancers: clinical and molecular characteristics and outcomes with mTOR inhibitors. PLoS One 2014;9:e89388.

43. Myers AP, Filiaci VL, Zhang Y, et al. Tumor mutational analysis of GOG248, a phase II study of temsirolimus or temsirolimus and alternating megestrol acetate and tamoxifen for advanced endometrial cancer (EC): An NRG Oncology/Gynecologic Oncology Group study. Gynecol Oncol 2016;141:43-8.

44. Sternberg CN, Davis ID, Mardiak J, et al. Pazopanib in locally advanced or metastatic renal cell carcinoma: results of a randomized phase III trial. J Clin Oncol 2010;28:1061-8.

45. Motzer RJ, Hutson TE, Glen H, et al. Lenvatinib, everolimus, and the combination in patients with metastatic renal cell carcinoma: a randomised, phase 2, open-label, multicentre trial. Lancet Oncol 2015;16:1473-1482.

46. Schlumberger M, Tahara M, Wirth LJ, et al. Lenvatinib versus placebo in radioiodine-refractory thyroid cancer. N Engl J Med 2015;372:621-30.

47. van der Graaf WT, Blay JY, Chawla SP, et al. Pazopanib for metastatic soft-tissue sarcoma (PALETTE): a randomised, double-blind, placebo-controlled phase 3 trial. Lancet 2012;379:1879-86.

48. Cortes JE, Kim DW, Pinilla-Ibarz J, et al. A phase 2 trial of ponatinib in Philadelphia chromosome-positive leukemias. N Engl J Med 2013;369:1783-96.

49. Wilson CH, McIntyre RE, Arends MJ, et al. The activating mutation R201C in GNAS promotes intestinal tumourigenesis in Apc(Min/+) mice through activation of Wnt and ERK1/2 MAPK pathways. Oncogene 2010;29:4567-75.

50. Cox AD, Fesik SW, Kimmelman AC, et al. Drugging the undruggable RAS: Mission possible? Nat Rev Drug Discov 2014;13:828-51.

51. Ideno N, Yamaguchi H, Ghosh B, et al. GNAS(R201C) Induces Pancreatic Cystic Neoplasms in Mice That Express Activated KRAS by Inhibiting YAP1 Signaling. Gastroenterology 2018;155:1593-1607.e12.

52. DiNardo CD, Stein EM, de Botton S, et al. Durable Remissions with Ivosidenib in IDH1-Mutated Relapsed or Refractory AML. N Engl J Med 2018;378:2386-2398.

53. MacKenzie ED, Selak MA, Tennant DA, et al. Cell-permeating alpha-ketoglutarate derivatives alleviate pseudohypoxia in succinate dehydrogenase-deficient cells. Mol Cell Biol 2007;27:3282-9.

54. Rohle D, Popovici-Muller J, Palaskas N, et al. An inhibitor of mutant IDH1 delays growth and promotes differentiation of glioma cells. Science 2013;340:626-30.

55. Janne PA, van den Heuvel MM, Barlesi F, et al. Selumetinib Plus Docetaxel Compared With Docetaxel Alone and Progression-Free Survival in Patients With KRAS-Mutant Advanced Non-Small Cell Lung Cancer: The SELECT-1 Randomized Clinical Trial. Jama 2017;317:1844-1853.

56. Adjei AA, Cohen RB, Franklin W, et al. Phase I pharmacokinetic and pharmacodynamic study of the oral, small-molecule mitogen-activated protein kinase kinase 1/2 inhibitor AZD6244 (ARRY-142886) in patients with advanced cancers. J Clin Oncol 2008;26:2139-46.

57. Manchado E, Weissmueller S, Morris JPt, et al. A combinatorial strategy for treating KRAS-mutant lung cancer. Nature 2016;534:647-51.

58. Infante JR, Somer BG, Park JO, et al. A randomised, double-blind, placebo-controlled trial of trametinib, an oral MEK inhibitor, in combination with gemcitabine for patients with untreated metastatic adenocarcinoma of the pancreas. Eur J Cancer 2014;50:2072-81.

59. Lito P, Saborowski A, Yue J, et al. Disruption of CRAF-mediated MEK activation is required for effective MEK inhibition in KRAS mutant tumors. Cancer Cell 2014;25:697-710.

60. Hochster HS, Uboha N, Messersmith W, et al. Phase II study of selumetinib (AZD6244, ARRY-142886) plus irinotecan as second-line therapy in patients with K-RAS mutated colorectal cancer. Cancer Chemother Pharmacol 2015;75:17-23.

61. Blumenschein GR, Jr., Smit EF, Planchard D, et al. A randomized phase II study of the MEK1/MEK2 inhibitor trametinib (GSK1120212) compared with docetaxel in KRAS-mutant advanced non-small-cell lung cancer (NSCLC)dagger. Ann Oncol 2015;26:894-901.

62. Zhu Z, Aref AR, Cohoon TJ, et al. Inhibition of KRAS-driven tumorigenicity by interruption of an autocrine cytokine circuit. Cancer Discov 2014;4:452-65.

63. Falchook GS, Lewis KD, Infante JR, et al. Activity of the oral MEK inhibitor trametinib in patients with advanced melanoma: a phase 1 dose-escalation trial. Lancet Oncol 2012;13:782-9.

64. Traynor K. Cabozantinib approved for advanced medullary thyroid cancer. Am J Health Syst Pharm 2013;70:88.

65. Hart CD, De Boer RH. Profile of cabozantinib and its potential in the treatment of advanced medullary thyroid cancer. Onco Targets Ther 2013;6:1-7.

66. Choueiri TK, Escudier B, Powles T, et al. Cabozantinib versus Everolimus in Advanced Renal-Cell Carcinoma. N Engl J Med 2015;373:1814-23.

67. Mazieres J, Zalcman G, Crino L, et al. Crizotinib therapy for advanced lung adenocarcinoma and a ROS1 rearrangement: results from the EUROS1 cohort. J Clin Oncol 2015;33:992-9.

68. Solomon BJ, Mok T, Kim DW, et al. First-line crizotinib versus chemotherapy in ALK-positive lung cancer. N Engl J Med 2014;371:2167-77.

69. Verstovsek S, Mesa RA, Gotlib J, et al. A double-blind, placebo-controlled trial of ruxolitinib for myelofibrosis. N Engl J Med 2012;366:799-807.

70. Verstovsek S, Mesa RA, Gotlib J, et al. The clinical benefit of ruxolitinib across patient subgroups: analysis of a placebo-controlled, Phase III study in patients with myelofibrosis. Br J Haematol 2013;161:508-16.

71. O'Sullivan JM, Harrison CN. JAK-STAT signaling in the therapeutic landscape of myeloproliferative neoplasms. Mol Cell Endocrinol 2017;451:71-79.

72. Kleppe M, Kwak M, Koppikar P, et al. JAK-STAT pathway activation in malignant and nonmalignant cells contributes to MPN pathogenesis and therapeutic response. Cancer Discov 2015;5:316-31.

73. Wagle N, Grabiner BC, Van Allen EM, et al. Activating mTOR mutations in a patient with an extraordinary response on a phase I trial of everolimus and pazopanib. Cancer Discov 2014;4:546-53.

74. Li H, Zeng J, Shen K. PI3K/AKT/mTOR signaling pathway as a therapeutic target for ovarian cancer. Arch Gynecol Obstet 2014;290:1067-78.

75. Laplante M, Sabatini DM. mTOR signaling in growth control and disease. Cell 2012;149:274-93.

76. Feng Z, Zhang H, Levine AJ, et al. The coordinate regulation of the p53 and mTOR pathways in cells. Proc Natl Acad Sci U S A 2005;102:8204-9.

77. Zaytseva YY, Valentino JD, Gulhati P, et al. mTOR inhibitors in cancer therapy. Cancer Lett 2012;319:1-7.

78. Janku F, Wheler JJ, Westin SN, et al. PI3K/AKT/mTOR inhibitors in patients with breast and gynecologic malignancies harboring PIK3CA mutations. J Clin Oncol 2012;30:777-82.

79. Janku F, Hong DS, Fu S, et al. Assessing PIK3CA and PTEN in early-phase trials with PI3K/AKT/mTOR inhibitors. Cell Rep 2014;6:377-87.

80. Lodish MB, Stratakis CA. Endocrine tumours in neurofibromatosis type 1, tuberous sclerosis and related syndromes. Best Pract Res Clin Endocrinol Metab 2010;24:439-49.

81. Hattori S, Ohmi N, Maekawa M, et al. Antibody against neurofibromatosis type 1 gene product reacts with a triton-insoluble GTPase activating protein toward ras p21. Biochem Biophys Res Commun 1991;177:83-9.

82. Janku F, Tsimberidou AM, Garrido-Laguna I, et al. PIK3CA mutations in patients with advanced cancers treated with PI3K/AKT/mTOR axis inhibitors. Mol Cancer Ther 2011;10:558-65.

83. Loi S, Michiels S, Baselga J, et al. PIK3CA genotype and a PIK3CA mutation-related gene signature and response to everolimus and letrozole in estrogen receptor positive breast cancer. PLoS One 2013;8:e53292.

84. Mackay HJ, Eisenhauer EA, Kamel-Reid S, et al. Molecular determinants of outcome with mammalian target of rapamycin inhibition in endometrial cancer. Cancer 2014;120:603-10.

85. Hortobagyi GN, Chen D, Piccart M, et al. Correlative Analysis of Genetic Alterations and Everolimus Benefit in Hormone Receptor-Positive, Human Epidermal Growth Factor Receptor 2-Negative Advanced Breast Cancer: Results From BOLERO-2. J Clin Oncol 2016;34:419-26.

86. Moynahan ME, Chen D, He W, et al. Correlation between PIK3CA mutations in cell-free DNA and everolimus efficacy in HR(+), HER2(-) advanced breast cancer: results from BOLERO-2. Br J Cancer 2017;116:726-730.

87. Wu R, Hu TC, Rehemtulla A, et al. Preclinical testing of PI3K/AKT/mTOR signaling inhibitors in a mouse model of ovarian endometrioid adenocarcinoma. Clin Cancer Res 2011;17:7359-72.

88. Koehler K, Liebner D, Chen JL. TP53 mutational status is predictive of pazopanib response in advanced sarcomas. Ann Oncol 2016;27:539-43.

89. Said R, Hong DS, Warneke CL, et al. P53 mutations in advanced cancers: clinical characteristics, outcomes, and correlation between progression-free survival and bevacizumab-containing therapy. Oncotarget 2013;4:705-14.

90. Schwaederle M, Lazar V, Validire P, et al. VEGF-A Expression Correlates with TP53 Mutations in Non-Small Cell Lung Cancer: Implications for Antiangiogenesis Therapy. Cancer Res 2015;75:1187-90.

91. Wheler JJ, Janku F, Naing A, et al. TP53 Alterations Correlate with Response to VEGF/VEGFR Inhibitors: Implications for Targeted Therapeutics. Mol Cancer Ther 2016;15:2475-2485.

92. Mertz JA, Conery AR, Bryant BM, et al. Targeting MYC dependence in cancer by inhibiting BET bromodomains. Proc Natl Acad Sci U S A 2011;108:16669-74.

93. Wyce A, Ganji G, Smitheman KN, et al. BET inhibition silences expression of MYCN and BCL2 and induces cytotoxicity in neuroblastoma tumor models. PLoS One 2013;8:e72967.

94. Delmore JE, Issa GC, Lemieux ME, et al. BET bromodomain inhibition as a therapeutic strategy to target c-Myc. Cell 2011;146:904-17.
